# Acheulean technology and landscape use at Dawadmi, central Arabia

**DOI:** 10.1371/journal.pone.0200497

**Published:** 2018-07-27

**Authors:** Ceri Shipton, James Blinkhorn, Paul S. Breeze, Patrick Cuthbertson, Nick Drake, Huw S. Groucutt, Richard P. Jennings, Ash Parton, Eleanor M. L. Scerri, Abdullah Alsharekh, Michael D. Petraglia

**Affiliations:** 1 Centre of Excellence for Australian Biodiversity and Heritage, Australian National University, Canberra, Australia; 2 McDonald Institute for Archaeological Research, University of Cambridge, Cambridge, United Kingdom; 3 Department of Geography, Royal Holloway, University of London, London, United Kingdom; 4 Department of Archaeology, Max Planck Institute for the Science of Human History, Jena, Germany; 5 Department of Geography, King’s College London, London, United Kingdom; 6 School of Archaeology, University of Oxford, Oxford, United Kingdom; 7 School of Natural Sciences and Psychology, Liverpool John Moores University, Liverpool, United Kingdom; 8 Department of Social Sciences, Oxford Brookes University, Oxford, United Kingdom; 9 Mansfield College, University of Oxford, Oxford, United Kingdom; 10 Department of Archaeology, King Saud University, Riyadh, Saudi Arabia; Institut Català de Paleoecologia Humana i Evolució Social (IPHES), SPAIN

## Abstract

Despite occupying a central geographic position, investigations of hominin populations in the Arabian Peninsula during the Lower Palaeolithic period are rare. The colonization of Eurasia below 55 degrees latitude indicates the success of the genus *Homo* in the Early and Middle Pleistocene, but the extent to which these hominins were capable of innovative and novel behavioural adaptations to engage with mid-latitude environments is unclear. Here we describe new field investigations at the Saffaqah locality (206–76) near Dawadmi, in central Arabia that aim to establish how hominins adapted to this region. The site is located in the interior of Arabia over 500 km from both the Red Sea and the Gulf, and at the headwaters of two major extinct river systems that were likely used by Acheulean hominins to cross the Peninsula. Saffaqah is one of the largest Acheulean sites in Arabia with nearly a million artefacts estimated to occur on the surface, and it is also the first to yield stratified deposits containing abundant artefacts. It is situated in the unusual setting of a dense and well-preserved landscape of Acheulean localities, with sites and isolated artefacts occurring regularly for tens of kilometres in every direction. We describe both previous and recent excavations at Saffaqah and its large lithic assemblage. We analyse thousands of artefacts from excavated and surface contexts, including giant andesite cores and flakes, smaller cores and retouched artefacts, as well as handaxes and cleavers. Technological assessment of stratified lithics and those from systematic survey, enable the reconstruction of stone tool life histories. The Acheulean hominins at Dawadmi were strong and skilful, with their adaptation evidently successful for some time. However, these biface-makers were also technologically conservative, and used least-effort strategies of resource procurement and tool transport. Ultimately, central Arabia was depopulated, likely in the face of environmental deterioration in the form of increasing aridity.

## Introduction

Understanding Pleistocene hominin adaptation and dispersal requires knowledge about the behaviour of species in different parts of their geographic range. Current information on Acheulean behaviour comes predominantly from the East African Rift Valley, the Levant, and western Europe, with arid areas to the east, such as the Arabian Peninsula, under-represented. Located between Africa, Europe, and South Asia, Arabia occupies a central position in the Acheulean occupied world, making it a key region for understanding hominin dispersal processes. Numerous surface Acheulean sites have in fact been found across the Arabian Peninsula from Yemen in the south to the Nefud desert in the north, and from the Red Sea coast in the west to Dhofar in the east [1–8], but there has been very little systematic assessment or controlled excavation. Moreover, the general paucity of preserved Pleistocene sedimentation in arid regions means that stratified Palaeolithic sites are scarce in many areas. Arid landscapes can however, be stable over long periods of time and have a high visibility of artefacts, enabling systematic surveys to reveal broad spatial patterns in hominin behaviour [9, 10]. The aim of this paper is to understand Acheulean hominin adaptation in Arabia using a combination of survey and excavation of stone artefacts.

Hominin use of their local landscape is critical to understanding how different regions were exploited and the extent to which they were able to adapt to environmental variation [11, 12]. Middle Pleistocene hominins were evidently able to occupy much of the Old World below a latitude of around 55 degrees. Their spread is an indication of the success of their adaptation, with *Homo* characterized as an ecologically dominant genus able to maintain a nutrient-rich diet [13]. *Homo* species are suggested to have been unusually adaptable to the environmental variability of the Pleistocene [14–16]. Despite this, their occupations in more hostile cold or arid areas were intermittent, being restricted to periods with more favourable environmental conditions [17, 18]. In comparison to their Middle Palaeolithic successors, Lower Palaeolithic hominins may have been less technologically innovative [19, 20] and had a more restricted use of the landscape [9, 21, 22]. In the Middle Palaeolithic of Arabia, hominins were climbing mountains to access high quality material for a variety of Levallois core types [23]; whereas there are no such upland sites in the Arabian Acheulean, with sites located near water sources and tools made on easier to access material [8].

In order to explore the hypothesis that Acheulean occupation was intermittent in marginal regions, we re-examine Saffaqah (206–76), the first stratified Acheulean site documented in Arabia and one of the largest Palaeolithic sites known in the Peninsula. The surrounding landscape is densely covered with Acheulean localities and the dearth of later cultural phases in the area [24] mean these have not been overwritten and disturbed. The scale of this landscape is large, with occurrences over tens of kilometres in every direction from Saffaqah 206–76 [25]. In conjunction with the stratified artefacts, this landscape perspective allows for holistic and diachronic evaluations of Acheulean hominin technological behaviours.

### Background

During the ‘Comprehensive Survey of the Kingdom’, conducted in the late 1970s, Acheulean sites were found in association with andesite dykes near the town of Dawadmi in central Saudi Arabia [1]. The most prominent andesite dyke is located close to the village of Saffaqah, and here the particularly large Acheulean site of 206–76 was discovered. Along this dyke dozens more Acheulean localities were recorded during the follow-up investigations of Norman Whalen and colleagues [26] and our own more recent investigations [25], but Saffaqah 206–76 remains the largest. Whalen and colleagues [26] undertook a systematic surface collection and subsequently excavated the site leading to the recovery of over 8,000 buried artefacts, making this the first stratified Acheulean site to be documented in Arabia [27].

**Fig 1:**
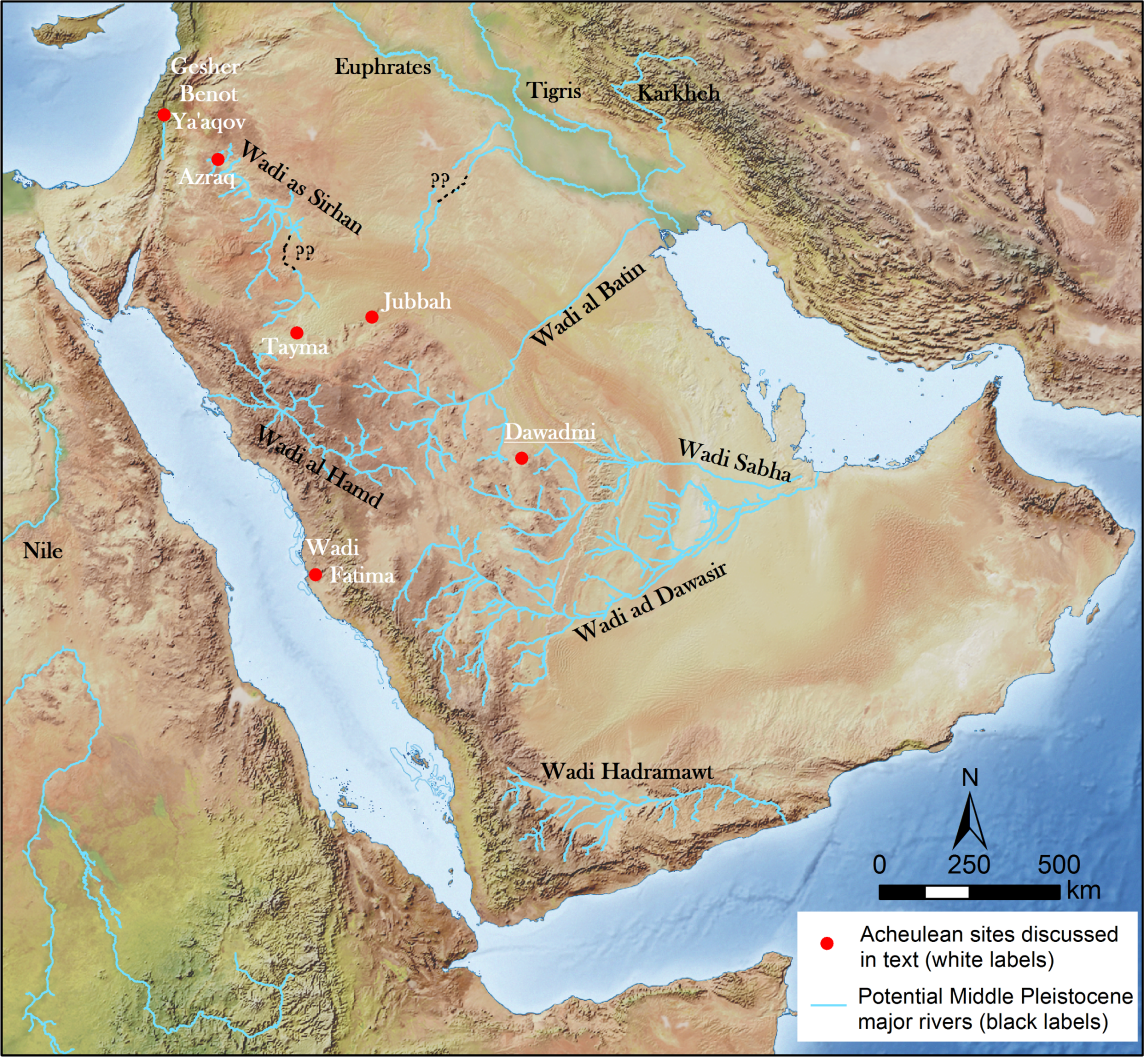
The location of Dawadmi in central Arabia, showing other areas of Acheulean discoveries mentioned in the text. Basemap reprinted with permission from Esri, ArcGIS, DigitalGlobe, GeoEye, i-cubed, USDA, USGS, Aex, Getmapping, Aerogrid, IGN, IGP swisstopo, and the GIS User Community under a CC-BY license, original copyright 2018.

**Fig 2:**
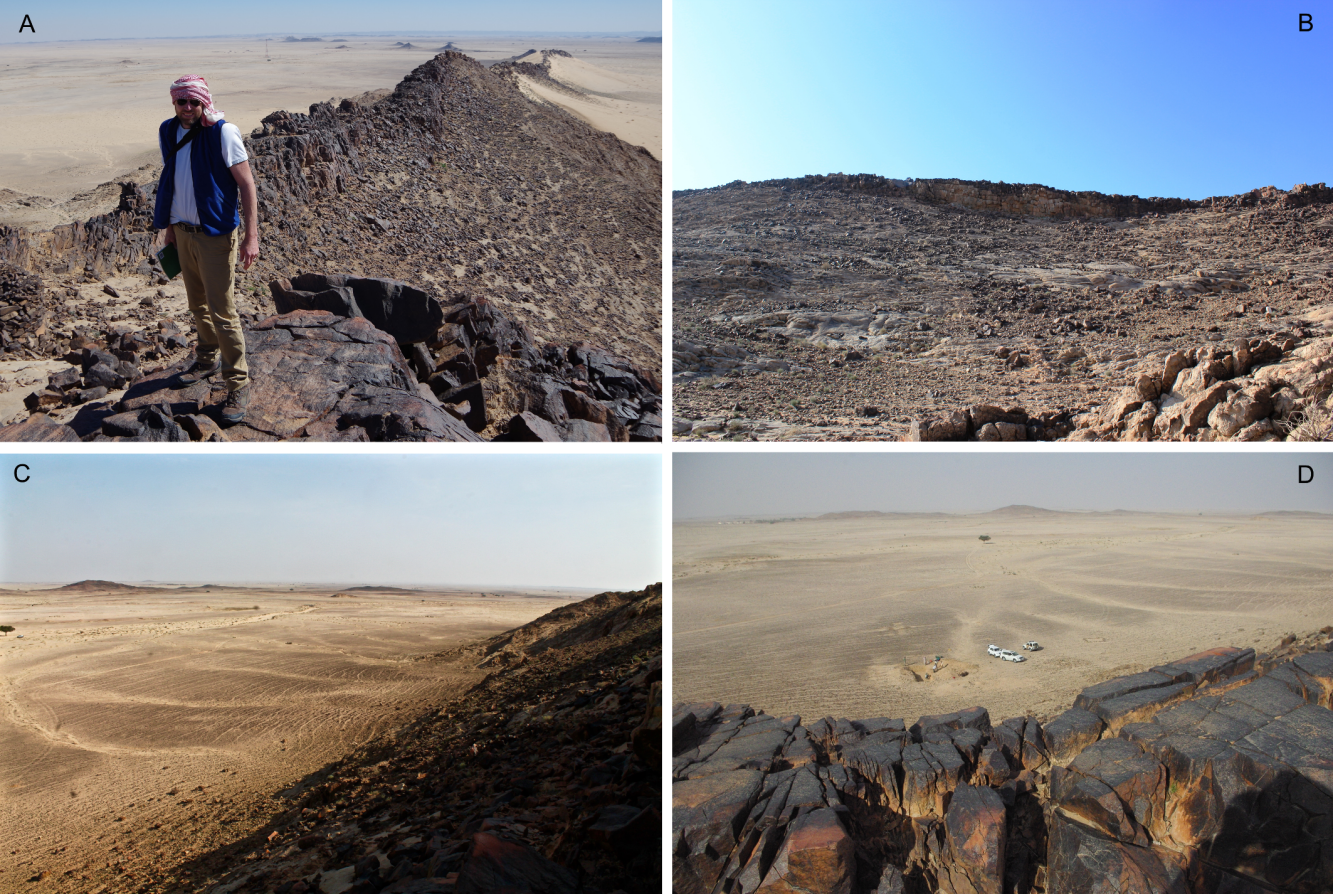
Four views of the Saffaqah dyke, showing the linear formation of the dyke and clasts eroding off the dyke (A); the blocky form in which the dyke weathers and clasts that have moved down the slope beneath the dyke (B) the terracettes that form on the stable lower flanks of the dyke (C); the primary unweathered dyke and the excavation next to the vehicles in the background (D).

Dawadmi is situated on the Nejd Plateau of the Arabian shield in the centre of the Peninsula (Fig 1) [25, 28]. The basement of the Nejd is Proterozoic igneous rock with granite occurring across much of the Dawadmi region. This basement is criss-crossed with intrusive felsic and mafic dykes [29], formed from the intrusion of magma from a shallow source through faults in the bedrock. When erosion exposes such dykes at the surface they form linear outcrops as they tend to be harder and therefore more resistant to erosion than the surrounding basement rock (Fig 2). Exposed dykes vary greatly in size, but generally form the most prominent topographic features on the landscape. Acheulean sites in the region are associated with andesite dykes that have a high topographic expression and low levels of fracturing (Fig 2): The larger dykes are usually constituted of the hardest and most homogenous material and produce the largest clasts, making it the most suited to being knapped into Acheulean tools. The flanks of the dykes and the area immediately surrounding them are covered in colluvium comprised of angular blocks of eroded material derived from the dyke ridges (Fig 2). These have been transported away from the dyke largely by surface creep which has led to the formation of well-defined terracettes [25] (Fig 2). Despite the abundance of dykes, there is low overall topographic relief around Dawadmi. The region is largely devoid of soft sediments except for the colluvium flanking the dykes and a thin veneer of aeolian sand in some areas, while the extreme aridity of the region means there is very little vegetation. Artefacts therefore usually occur on the surface and are highly visible.

The Saffaqah dyke rises higher than any other in the region, up to ~60 m above the surrounding plain. According to our surface surveys, Saffaqah 206–76 is both the largest site in extent and by far the densest in artefact numbers, having an estimated surface assemblage size nearly an order of magnitude greater than the next largest site in the Dawadmi region [25]. It is the largest known Acheulean site anywhere in the Arabian Peninsula.

The vast majority of artefacts at Saffaqah were made of andesite, however quartz, rhyolite, and granite were also used occasionally. Fractured quartz seams outcrop frequently on the flat plain to the north of the Saffaqah dyke, while a quartz jebel (rocky hill), with clasts large enough to make handaxes, occurs some 7 km to the east of Saffaqah 206–76. Granite boulders can be found at the base of a dyke 5 km to the east of Saffaqah 206–76. The nearest large rhyolite outcrop is found on a dyke around 5 km to the west of Saffaqah 206–76.

Small relict drainage channels (wadis), with a maximum depth of ~2 m cross the Dawadmi region [25]. Reconstruction of palaeodrainage, according to the methods detailed in Breeze et al. [30], demonstrates that Saffaqah sits at the interface between two major periodically active river systems: the Wadi al Batin and the Wadi Sabha, which flowed into the northern and southern ends of The Gulf respectively (Fig 1) [28]. In fact, Saffaqah 206–76 sits within 1 km of the drainage divide between these two river systems, as two of the wadis in our intensive study area eventually drain into the Wadi al Batin and two into the Wadi Sahba. Processes such as avulsion are common to high energy channels within medial-distal reaches, but drainage at Saffaqah consists of first order minor headwater streams with very small catchment areas that are too small to have altered the landscape substantially, or led to the significant redistribution of artefacts [25]. On the downstream (northern) side of the dyke where all the Acheulean sites associated with wadis are situated, the wadis are notably constrained in their positions as they flow between gaps in the dyke and then their channels are incised into the bedrock. This, combined with very low relief, has led to channel stability throughout the survey area, with no visible signs of disturbance or sediment aggradation in areas adjacent to the wadis.

**Fig 3:**
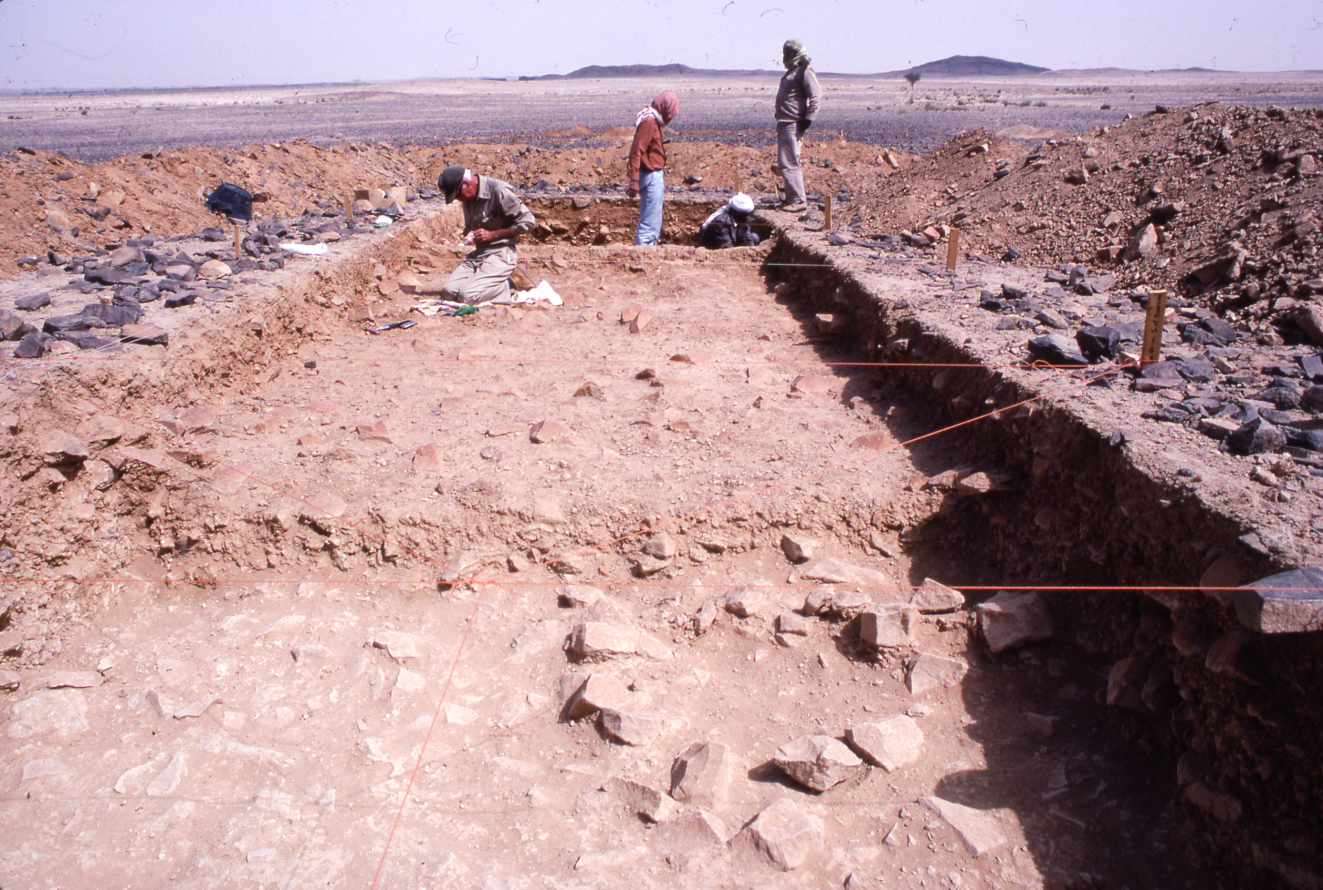
Whalen and colleagues’ trench during excavation, looking north away from the dyke. Note that the surface clasts have a blue patina, those at the level of the kneeling person (Norman Whalen) have a reddish patina due to iron staining, and those in the foreground at the level below are khaki coloured.

In their first season at Dawadmi, Whalen and colleagues noted the high concentration of artefacts on the surface at Saffaqah 206–76 and decided to conduct a systematic surface collection. A 30x30 m grid was divided into 3x3 m squares and all 3,235 identified artefacts in this grid were collected. Over two seasons in 1982 and 1983 Whalen and colleagues returned to the site to excavate (Fig 3). Three squares of the original survey grid produced only very shallow sediments, but a further 3 squares were more productive resulting in the main 11x3 m trench at the site. The trench runs N-S perpendicular to the dyke, and begins ~15 m from the break of slope that rises up to the dyke [26, 27]. Whalen et al. [27] reported that a total of 8,395 artefacts were recovered from the 33 m^2^ excavation, which extended to a depth of ~1.5 m. The excavation was conducted in 10 cm arbitrary spits, and no stratigraphic changes were recognised within the trench. Whalen et al. did, however, piece plot all artefacts as well as recording their material, dimensions, and type. This created a valuable archive of data which we were able to use in conjunction with our renewed work at the site. Calcrete was present on the clasts from 30 cm downwards, while sterile sediment was reached at depths of 1.33–1.49 m, and weathered granite bedrock at 1.62 m. Uranium-thorium ages on calcrete adhering to artefacts suggested that some were more than 200,000 years old, making this the oldest dated archaeological site in the peninsula at the time [27]. In 2014 the Palaeodeserts Project reinvestigated Saffaqah 206–76 with a view to refining the chronology and formation processes, assessing the stone tool technology, and situating the site within its landscape context.

## Methods

Permission to carry out the study was granted by HRH Prince Sultan bin Salman, President of the Saudi Commission for Tourism and National Heritage (SCTH) and Professor Ali I Al-Ghabban, Vice President. The individuals depicted in this manuscript (or their nearest living relatives) have given written informed consent (as outlined in the PLOS consent form) to publish their images.

### Systematic survey

**Fig 4:**
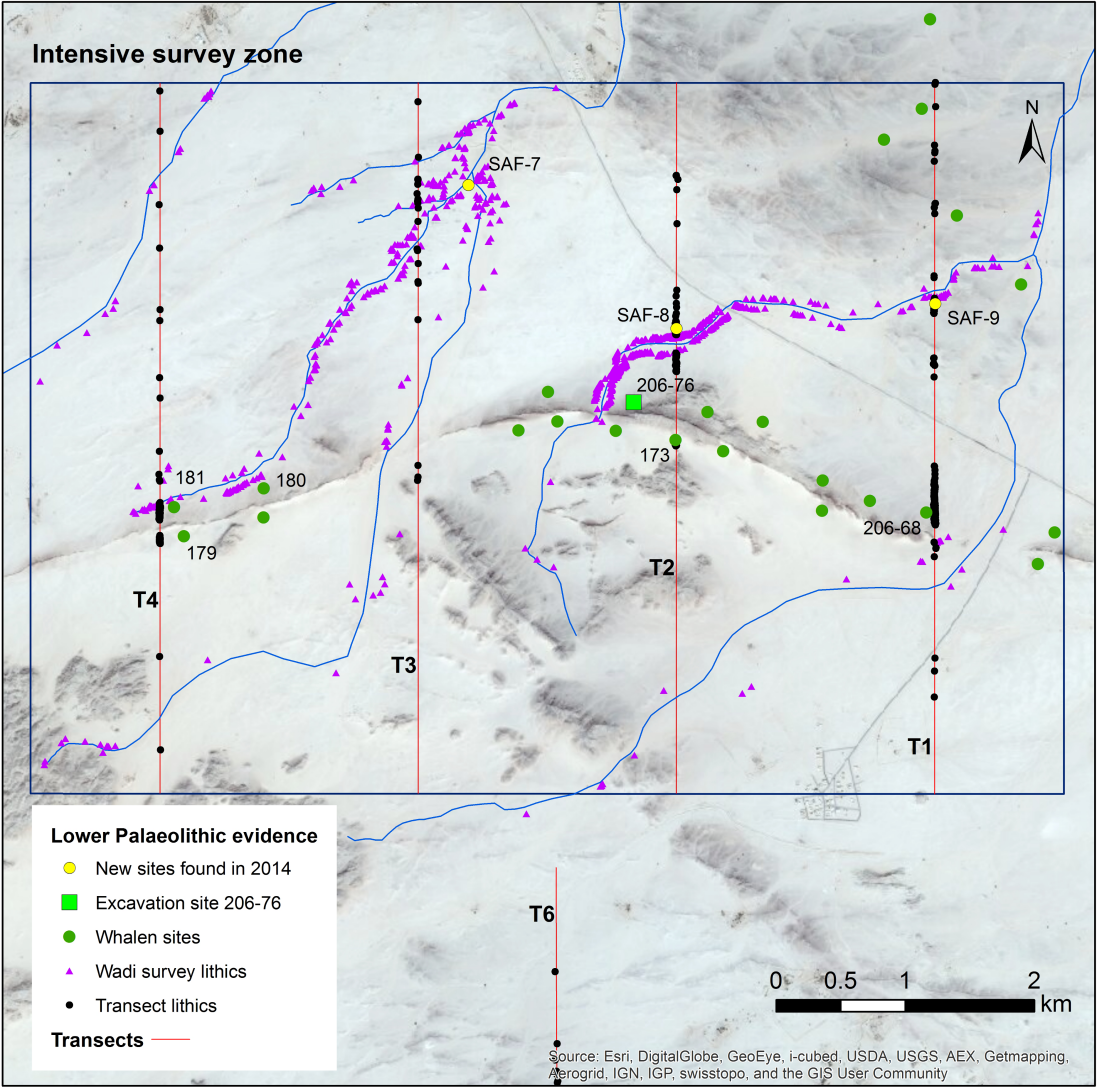
The intensive survey area along the Saffaqah dyke, showing both Whalen’s sites and new sites discovered during the survey, as well as individual artefact locations. Base layers reprinted with permission from Esri, ArcGIS, DigitalGlobe, GeoEye, i-cubed, USDA, USGS, Aex, Getmapping, Aerogrid, IGN, IGP swisstopo, and the GIS User Community under a CC-BY license, original copyright 2018.

To situate Saffaqah 206–76 in its archaeological landscape, we conducted a systematic survey of the surrounding area [20]. Straight-line transects were walked N-S, approximately perpendicular to the dyke, in order to understand how artefacts varied with distance from source. Four transects 10 m wide and 5.5 km long, were walked across the dyke at regularly spaced intervals in an 8 x 5.5 km area centred on Saffaqah 206–76 (Fig 4). In addition, 10 m wide transects were walked on either side of wadis that flow past the dyke to investigate the association of artefacts and drainage [20]. These transects were walked in teams of four and every artefact encountered along them had its material, typology, dimensions, and location recorded.

All artefacts from both the survey and the excavation are housed in the National Museum of Saudi Arabia in Riyadh. Specimen numbers for survey artefacts are: t1fx; t2fx; t3fx; t4fx; w1fx; w2fx; w3fx; w4fx (where ‘t’ refers to a straight line transect, ‘w’ refers to a wadi transect, and ‘f’ refers to a find with a suffix numeral ‘x’). Excavated artefacts do not have individual identification numbers.

### The excavation and stratigraphy of Saffaqah 206–76

**Fig 5:**
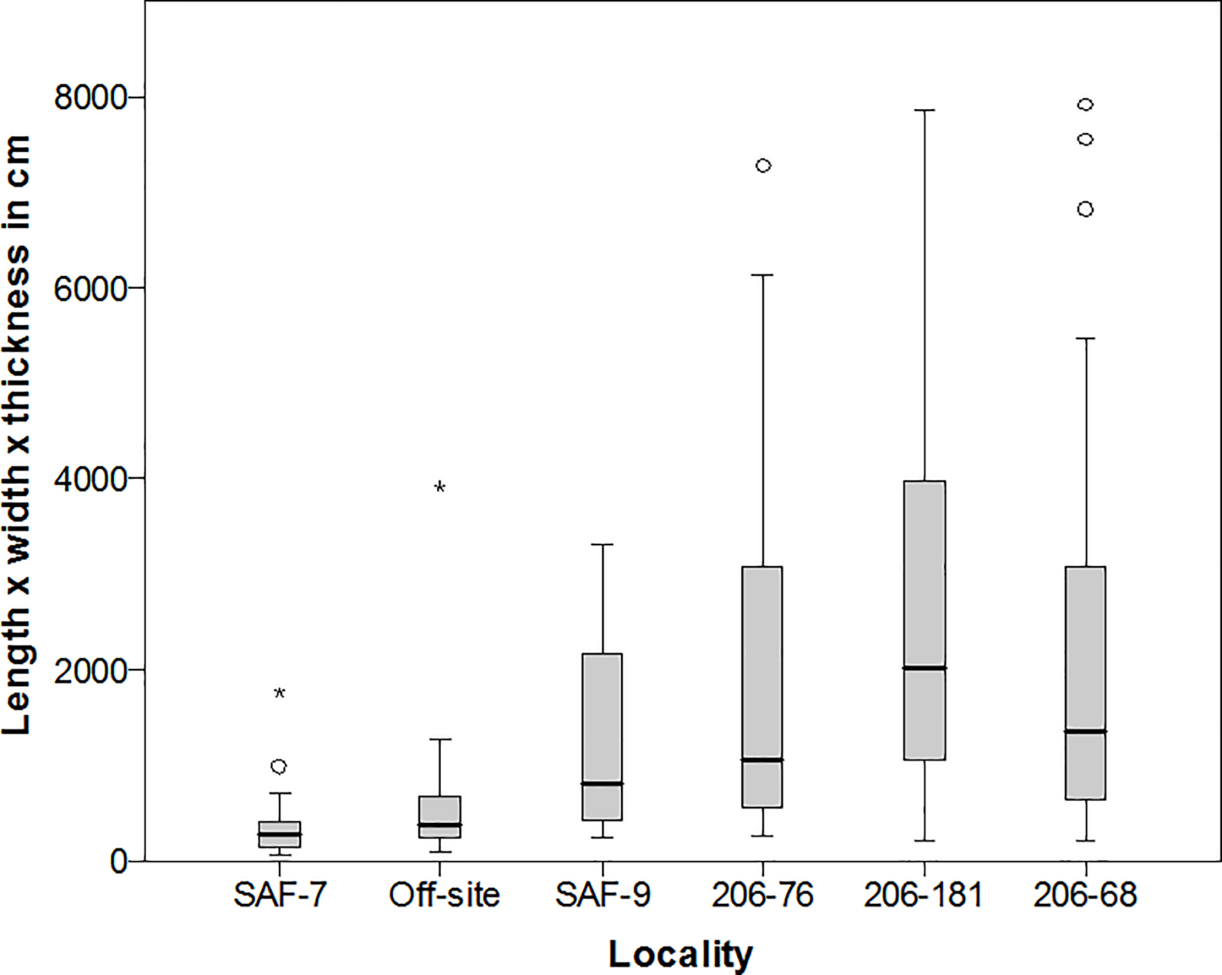
Plan of the Whalen et al. and Palaeodeserts trenches, showing Whalen’s three excavation units (S, T, and U) and the section of his excavation from which lithics were sampled for depth analysis in the present study.

To assess the stratigraphic and behavioural sequence at Saffaqah 206–76 we conducted our own excavation adjacent to the Whalen trench. This began with clearing out Whalen’s collapsed trench, and then cleaning and recording the well-preserved eastern section. In the southern portion of Whalen’s trench (Whalen’s 3 x 3 m unit U), the archaeological deposits narrow and pinch out upslope towards the base of the dyke. In the northern portion of the trench (Whalen’s 3 x 3 m unit S), the basal sterile deposits rise more steeply than the surface so that the archaeological deposits also become thinner and pinch out in this direction. Several sondages at approximately 10 m intervals running north downslope from Whalen’s trench confirmed increasingly attenuated stratigraphic sequences, and lacked the buried artefacts of Whalen’s trench. In the central part of Whalen’s trench, unit T (3 x 5 m), there was a shallow depression, which has infilled to give a thicker stratigraphic sequence. We placed an excavation trench measuring 2.5 x 1.5 m adjacent to the eastern side of Whalen’s unit T, in the northern half of the unit (Fig 5). It was aligned parallel to the trench of Whalen et al. [27], next to one of the original wooden grid pegs still visible on the surface. During the excavation of the first context, the remains of the equivalent grid peg were discovered 2.5 m away in line with the southern edge of the newly excavated trench.

The excavation was undertaken using the single context method. Artefacts were generally large enough to be picked out during excavation, but the dry sediment was also hand screened for smaller pieces. Little evidence for bioturbation was observed in the sediment, with compaction as well as clast size and frequency precluding burrows. There were however occasional roots up to 1.5 cm thick (less than the maximum dimension of the smallest artefacts). Filled 10 litre buckets were counted by context for artefact density calculations. Seven stratigraphic units (Layers A-F) were differentiated in the exposed section, which corresponded to 11 contexts identified during excavation (Layer B was subdivided into four contexts and Layer D into two contexts).

To explore the technology of the material excavated in the 2014 season, all lithics were typed and measured from contexts 1–3 (Layer A and the upper part of Layer B) and from the first spit of context 10 (the upper part of Layer E; this context was divided into four spits as it was particularly rich). In addition, a sample of 50 cores from the surface of Saffaqah 206–76 were analysed to assess core reduction sequences employed at the site. These cores were selected by one individual walking transects across the site and measuring all cores >50 mm in maximum dimension.

### Artefact distribution in the Whalen excavation

To further characterize the depositional and behavioural sequence at the site we analysed the size and spatial distribution of artefacts in the Whalen excavation. Whalen and colleagues piece-plotted, typed, and measured the maximum dimensions of each artefact they excavated from 206–76, but they produced no distribution maps. Whalen et al. [27] reported 8,395 excavated artefacts, though when we used the Whalen archive to digitise his artefact sheets, we found 9,491 entries. After eliminating ‘chunks’ and pieces of degrading granite bedrock we were left with 7,200 entries. We then regrouped Whalen’s original typology into a simpler series of types: cores, flakes, retouched flakes, shaped bifaces, and other core-tools (choppers and picks).

When Whalen and colleagues recorded the location of artefacts they measured depth below the ground surface, rather than a consistent, external benchmark. We used a total station to record the stratigraphy of the earlier trench along the eastern and southern walls, highlighting the significant slope of the ground surface. In order to integrate the 3D artefact data with our recording of the sloping ground surface and stratigraphy, reference points were added to the artefact dataset with height values of 0 m, relating to the original ground surface. The artefact dataset was then rotated to align these reference points to the actual ground-level dataset, measured by total station. The artefacts were then attributed to layers by projecting the measured stratigraphy across the width of the trench. The stratigraphy appeared broadly flat across the trench with neither the western wall of our excavation, nor the western wall of Whalen’s excavation differing significantly from the eastern wall.

A very limited number of artefacts were attributed to Layer F, which was identified as archaeologically sterile in our own excavations, and we therefore include them with finds attributed to Layer E for analysis (referred to as E/F below). Similarly, artefact density in Layer A is also low and preliminary statistical testing identified no significant differences in either composition or size variables with the underlying horizon. As a result, artefacts from Layer A and B are combined for detailed analyses (referred to as A/B below).

Multiple pair-wise Fisher’s Exact tests were conducted to evaluate whether significant differences occurred in the typological composition of each layer. Metric variability within artefact classes was evaluated at a pairwise level using Wilcox tests. A Benjamini-Hochberg correction was used to modify the p-value appropriately for multiple pairwise comparisons. Metric variables evaluated include artefact length, width, and thickness for all artefacts, estimated volume for cores, bifaces, and core-tools, elongation (length/width) for flakes, and refinement (thickness/width) for bifaces.

## Results

### Saffaqah intensive survey

**Fig 6:**
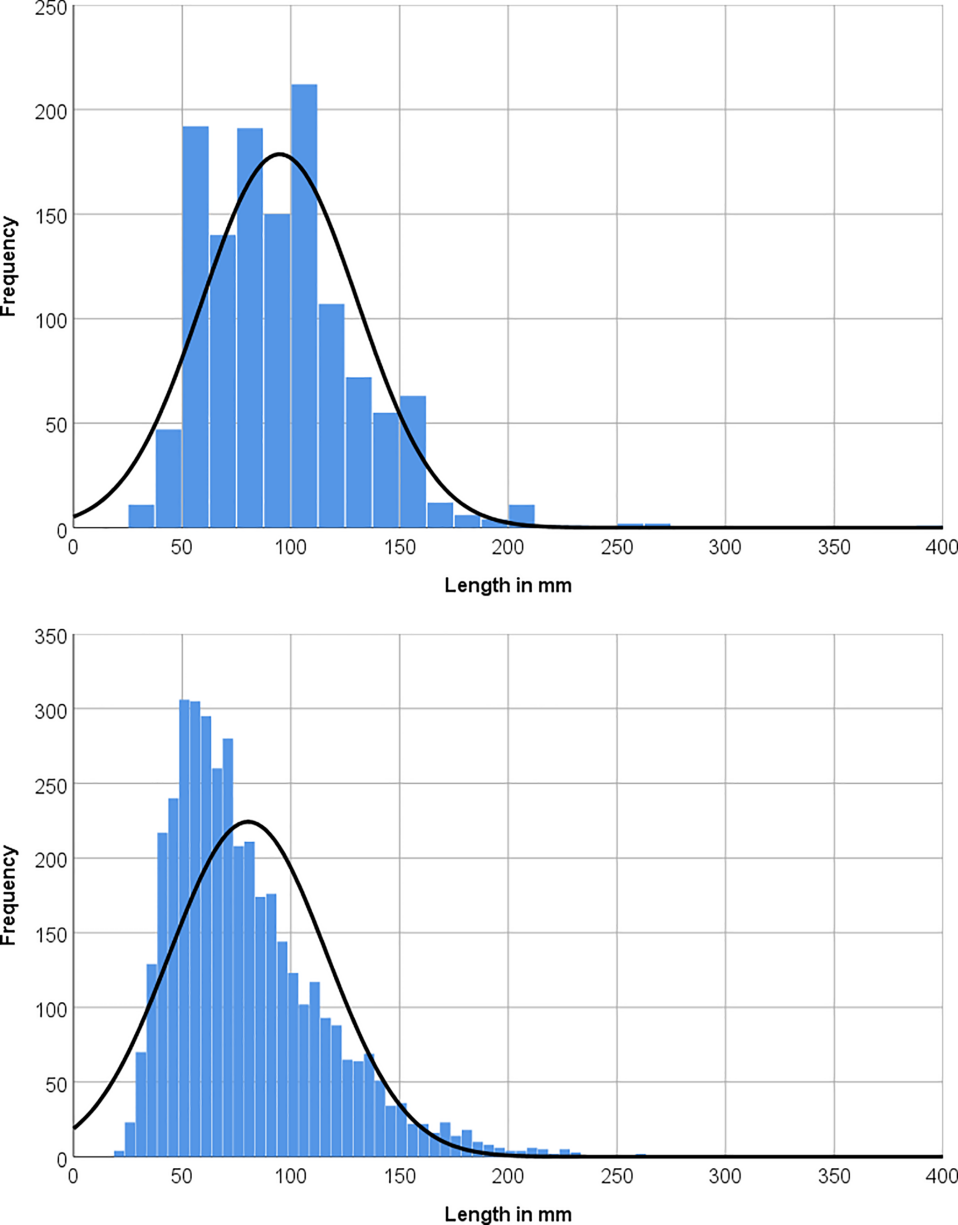
Histograms of axial flake length for artefacts on the intensive survey (above) and in the excavation of Whalen and colleagues at Saffaqah 206–76 (below).

Artefacts encountered during the Saffaqah survey were generally rather large, with the great majority of flakes over 50 mm long and reaching a maximum length of 390 mm (Fig 6). While this large size speaks to the general character of the Acheulean technology in the region, it is also likely that some of the smaller flakes have been destroyed or rendered unrecognisable through wind erosion.

The archaeological localities within our intensive survey area can be divided into three broad categories: localities associated with the dyke; localities associated with wadis; and off-sites of individual isolated finds [25] (Fig 4). These locality types are distinguished not only by their size and location but also by the typological and material composition of the lithics (Table 1). Dyke localities such as 206–76, 206–68, and 206–181 contain lithics nearly exclusively made on andesite, with a high proportion of flakes and early stage bifaces (elongate bifacially worked clasts that appear to have been abandoned before they were made into handaxes, abbreviated as ESB). Off-sites include a high proportion of finished bifaces and a higher proportion of non-andesite artefacts. Wadi localities are similar to off-sites in having high proportions of handaxes and cleavers, as well as non-andesite material, but they contain numerous artefacts over large areas like the dyke localities [25]. Given the small size and stability of the wadis, the introduction of generally larger handaxes rather than smaller flakes, and artefacts of geologies not found upstream, these patterns must be behavioural rather than taphonomic.

**Table 1:** The composition of a selection of Saffaqah surface assemblages recorded during the intensive survey. Note that two hammerstones were recorded from 206–68. Localities 206–76, 206–68, and 206–181 are associated with the dyke, while SAF-7 and SAF-9 are associated with wadis.

Locality	Andesite	Other	N	Cores	Flakes (inc. ≥100 mm)	Flakes ≥100 mm	Retouched	Early Stage Bifaces	Bifaces (inc. cleavers)	Cleavers
**206–68**	416 (99.5%)	2 (0.5%)	418	44 (10.6%)	260 (62.1%)	159 (38%)	18 (4.3%)	48 (11.5%)	46 (11%)	4 (1%)
**206–76**	293 (99.3%)	2 (0.7%)	295	35 (11.9%)	201 (68.1%)	105 (35.6%)	8 (2.7%)	11 (3.7%)	40 (13.6%)	1 (0.3%)
**206–181**	140 (97.2%)	4 (2.8%)	144	20 (13.9%)	76 (52.8%)	41 (28.5%)	4 (2.8%)	15 (10.4%)	29 (20.1%)	1 (0.7%)
**Off-sites**	242 (82.6%)	51 (17.4%)	293	31 (10.6%)	117 (39.9%)	34 (11.6%)	9 (3.1%)	11 (3.7%)	125 (42.7%)	5 (1.7%)
**SAF-7**	175 (81.9%)	39 (18.1%)	214	26 (12%)	61 (28.6%)	17 (7.9%)	6 (2.3%)	0	121 (55.8%)	3 (1.4%)
**SAF-9**	58 (96.5%)	2 (3.5%)	60	6 (10%)	30 (50%)	5 (8.3%)	1 (1.7%)	2 (3.3%)	21 (35%)	2 (3.3%)

Lithic production was one of the primary activities undertaken at workshop (manufacturing) localities at the base of the dyke; with a focus on making bifaces indicated by many elongate bifacially worked cobbles and large flake blanks. Table 1 indicates that some material was transported away from these dyke localities by hominins, in particular bifaces, though cores and flakes were also removed. These were used at varying locations on the landscape where they were often discarded in isolation, but a high proportion were curated and eventually deposited at localities beside wadis. The greater variety of materials at the wadi localities suggests these were used repeatedly, with hominins transporting material to them from multiple sources.

**Fig 7:**
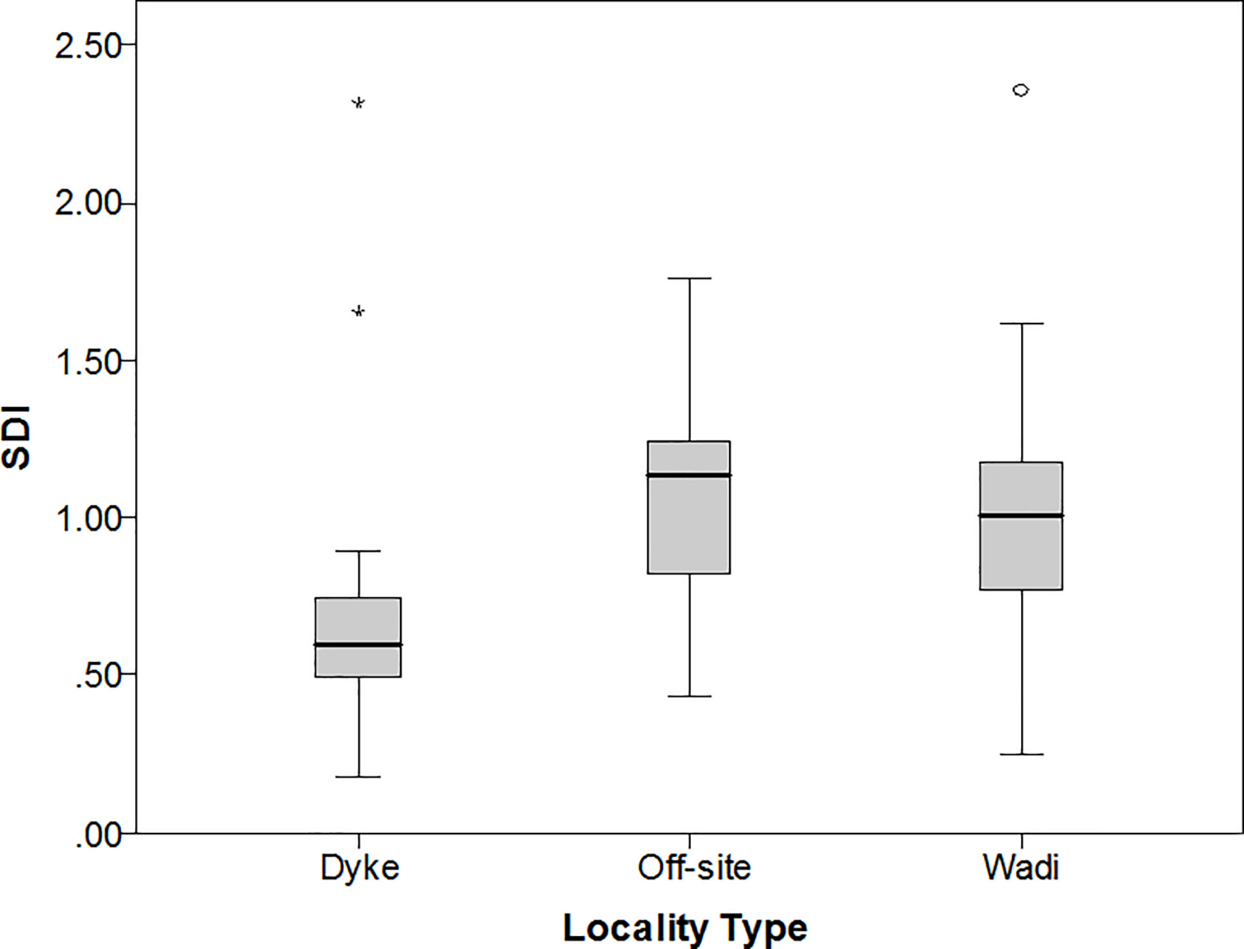
Boxplot of estimated core volume for selected Saffaqah localities, showing that localities associated with the dyke have the largest cores. Note that the very largest cores from 206–76 are too large to show at this scale.

Cores occurred in similar proportions across locality types (Table 1), however many of those from dyke localities were much larger than those from the wadi localities and off-sites (Fig 7). An ANOVA test showed there was significant variation in estimated core volume (length x width x thickness) between site types at the P = 0.007 level (N = 151, F = 3.344). The cores transported away from the dyke sites were likely for the creation of small sharp flakes, rather than for making large biface blank flakes. Indeed, the proportion of flakes under 100 mm in length is larger at sites away from the dykes than those at the dykes (Table 1).

**Fig 8:**
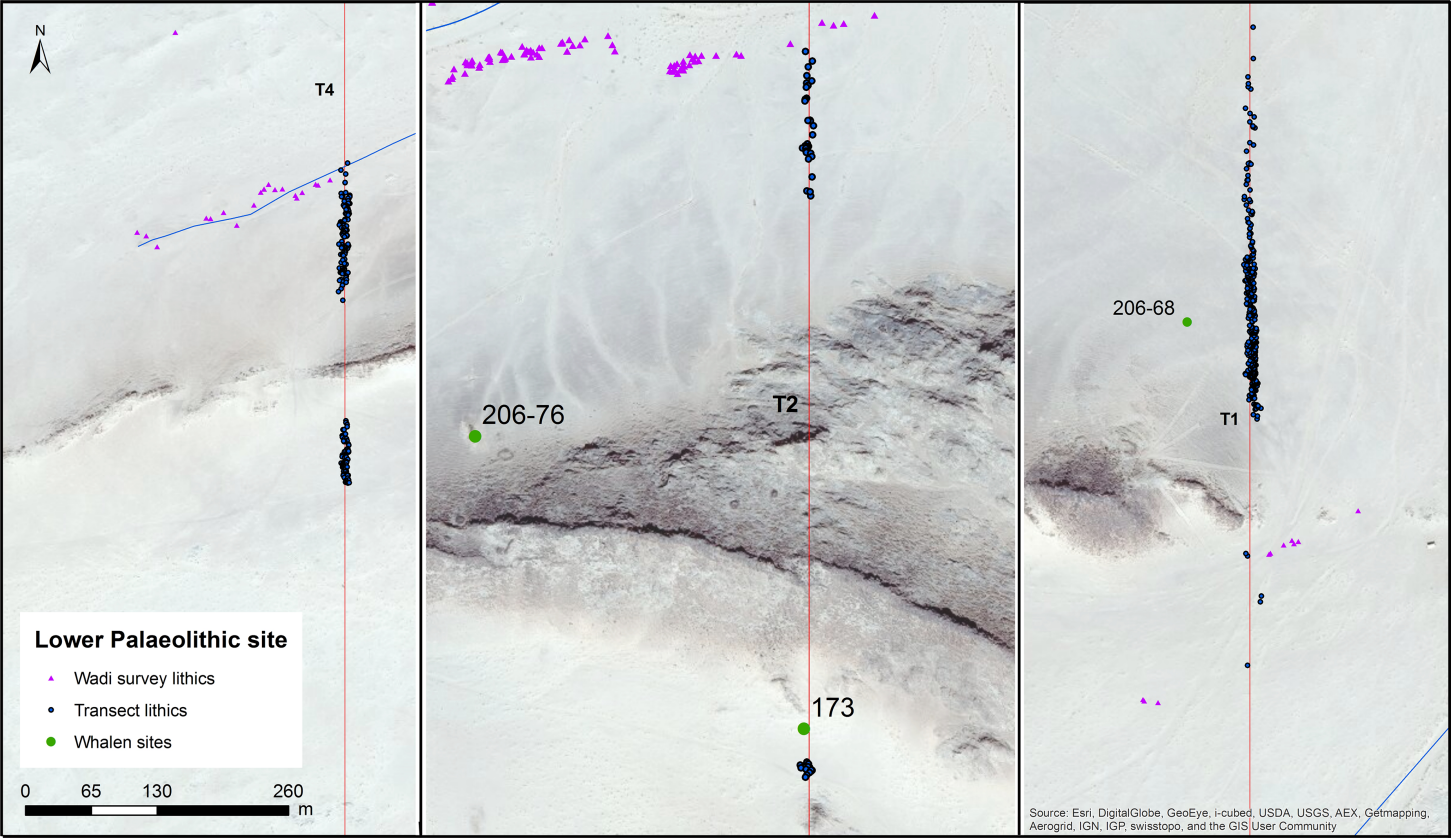
Boxplot of the scar density index (scars per square inch) for a sample of 84 Saffaqah bifaces (including early stage pieces), showing that bifaces away from the dyke are more reduced than those near the dykes. Sites where a wadi flowed past a dyke were classed as dyke sites. Base layers reprinted with permission from Esri, ArcGIS, DigitalGlobe, GeoEye, i-cubed, USDA, USGS, Aex, Getmapping, Aerogrid, IGN, IGP swisstopo, and the GIS User Community under a CC-BY license, original copyright 2018.

To trace the life history of bifaces between these site types we used the scar density index, a measure of lithic reduction intensity [31]. A sample of complete bifaces (including early stage pieces) with the most discernible flake scar boundaries that were collected during the survey were selected to cover the range of variation in material and form. These were scanned using a NextEngine laser scanner to measure their surface area. Scar densities were plotted for dyke sites, off-sites, and wadi sites (Fig 8). An ANOVA test showed that the pattern of higher scar densities at off-sites and wadi sites in comparison to dyke sites was significant (N = 84, F = 4.653, P = 0.012). This indicates that bifaces transported away from the workshops at the dykes undergo significant reduction, either through initial shaping to a particular form or through resharpening. To test between these two scenarios the relationship between size and reduction intensity was examined for bifaces transported away from the dykes. If smaller bifaces had been reduced more this is consistent with resharpening; on the other hand, if smaller bifaces were simply made on smaller blanks this suggests that they were not resharpened. A regression analysis of scar density and weight for the wadi and off-site bifaces showed no significant relationship between the two variables, suggesting that resharpening was not a significant factor in this assemblage and that variation in size was instead the result of initial blank size (df = 64, F = 0.055, P = 0.815, R^2^ = 0.001). Given the abundance of material suitable for knapping in this landscape there would have been little need to prolong the use-life of tools through resharpening.

**Fig 9:**
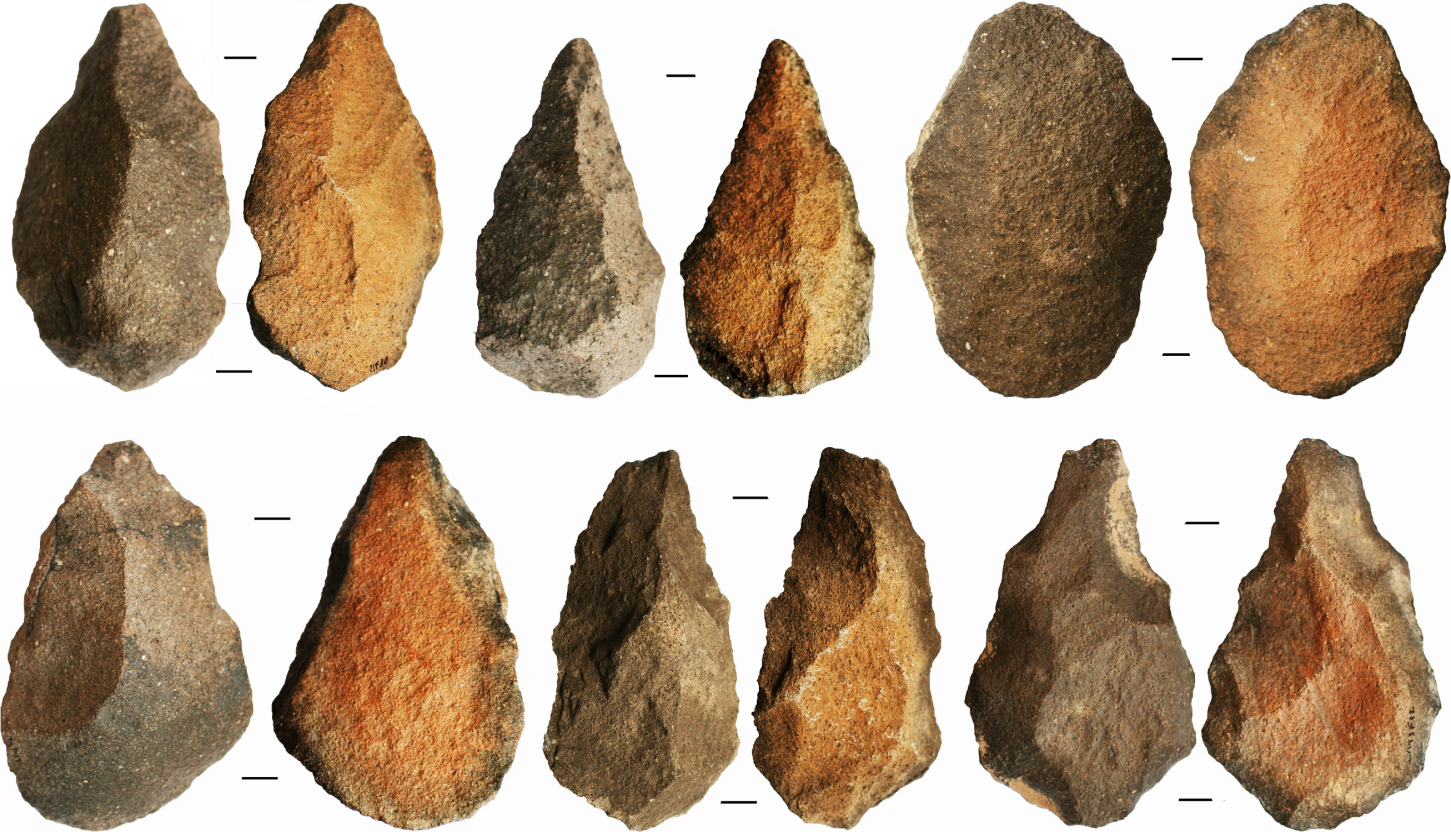
**The transects crossing the Saffaqah dyke at, from left to right, the western, central, and eastern portions of the dyke.** Note that artefacts stop abruptly on the approach to the dyke.

**Fig 10:**
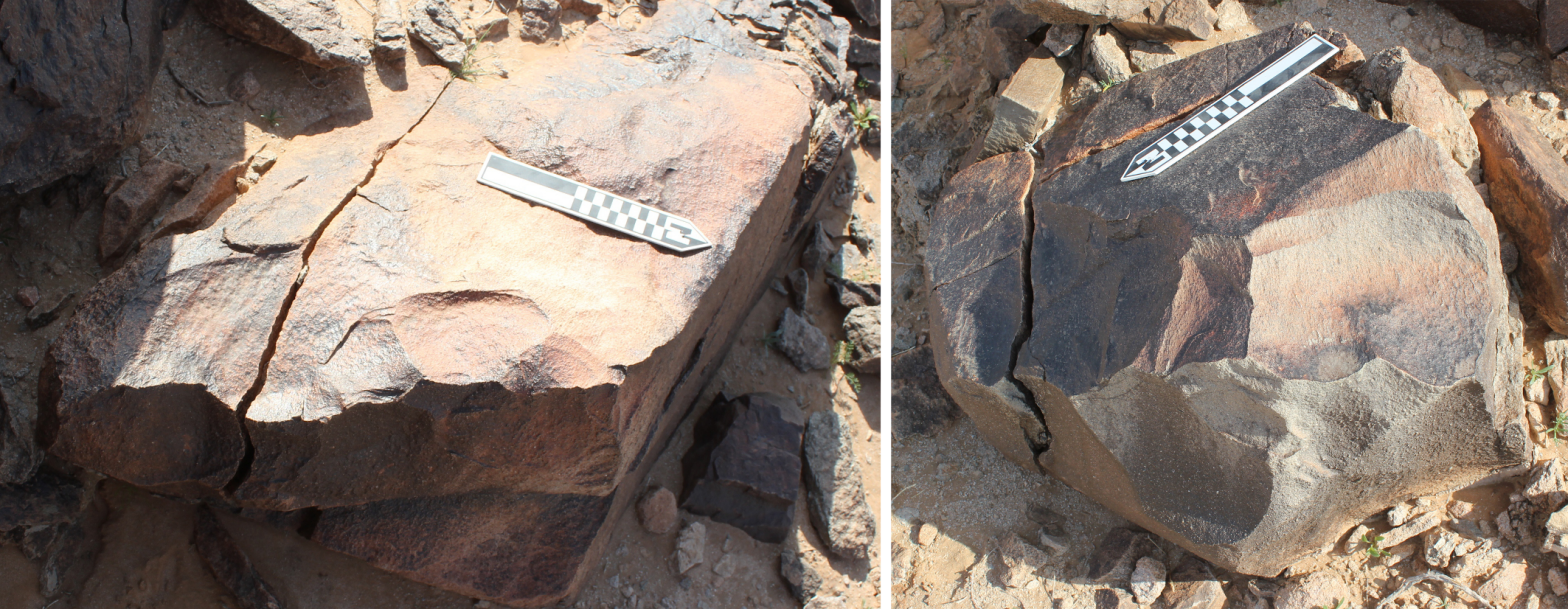
A selection of bifaces from Saffaqah showing pale purple to dark brownish grey patination on the upper exposed surface, and orange patination on the lower protected surface.

**Fig 11:**
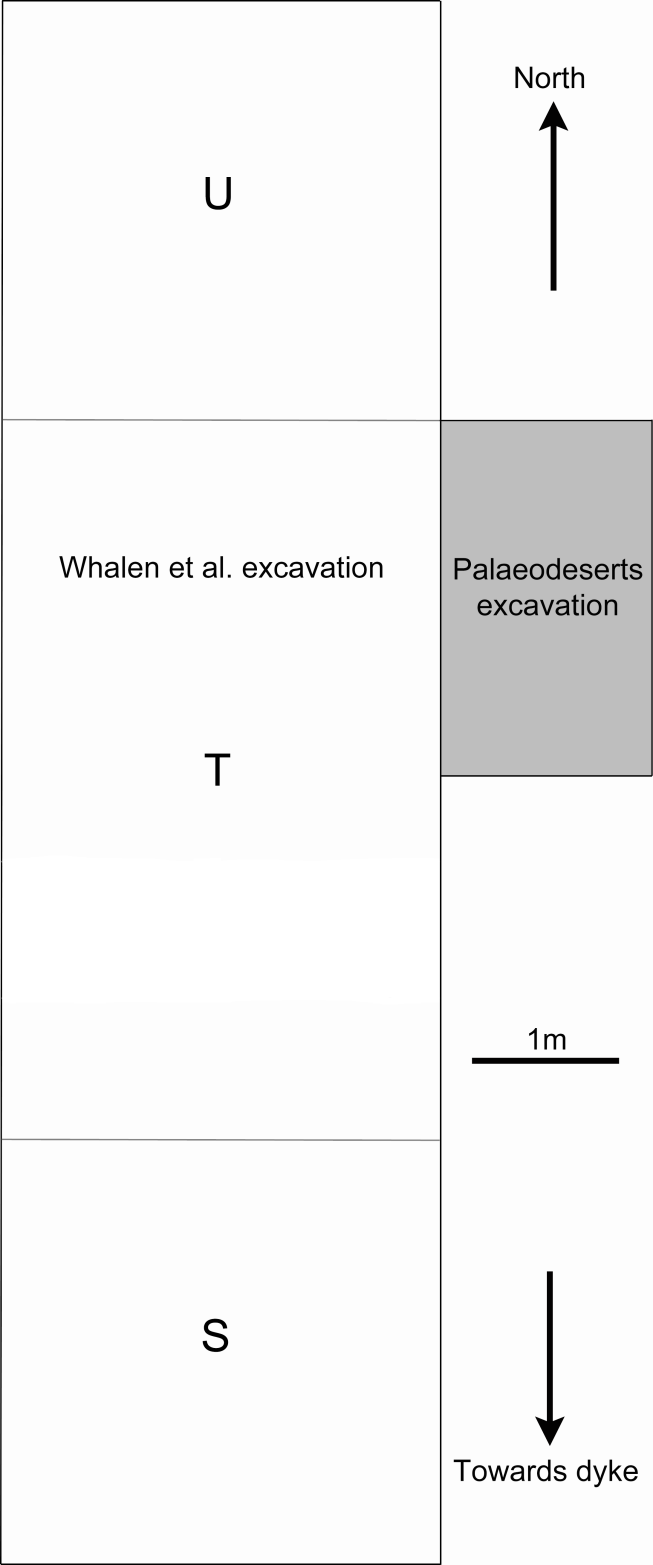
Two large blocks of andesite at the base of the dyke slope used as giant bifacial cores at Saffaqah 206–76.

Whalen et al. [26] reported two low density localities on small terraces on the Saffaqah dyke just to the east of 206–76, each apparently associated with a now extinct small water course. However, in repeated attempts to relocate these localities we did not observe any artefacts on these terraces. Furthermore, on the three instances where transects crossed the dyke, we did not observe any artefacts on the dyke or its flanks, despite numerous artefacts on either side (Fig 9). This pattern might be a taphonomic effect, resulting from the downward movement of artefacts on the dyke in the millennia since their discard by hominins. However, this hypothesis is belied by a number of factors. The dyke slopes are formed of terracettes (Fig 2, also see figures 3 and 9 in [25]) running along the slope. These features form when soil expands and contracts during humid-dry seasonal cycles [32]. The climate of Dawadmi is too arid for these to be forming currently, therefore the fact that they are still so visible indicates the slopes have been stable for a long time and not subject to significant colluvial movement. Artefacts do not appear to have moved downslope to flat areas as Saffaqah 206–76 has the highest density of artefacts near the excavation, yet it has the steepest slope of any of the dyke sites we investigated. The artefacts from Saffaqah, particularly those near the dykes, have a very different patina on their upper and lower surfaces indicating they have been lying in their present positions for a long time (Fig 10). Hominins were exploiting extremely large blocks of andesite that had detached from the dyke and fallen to the base (Fig 11), so we would expect them to flake the dyke itself if they were climbing the dyke to procure lithic material. However, we found no evidence for primary flaking of the dyke.

### The 2014 excavation of Saffaqah 206–76 and the stratigraphy of the site

**Fig 12:**
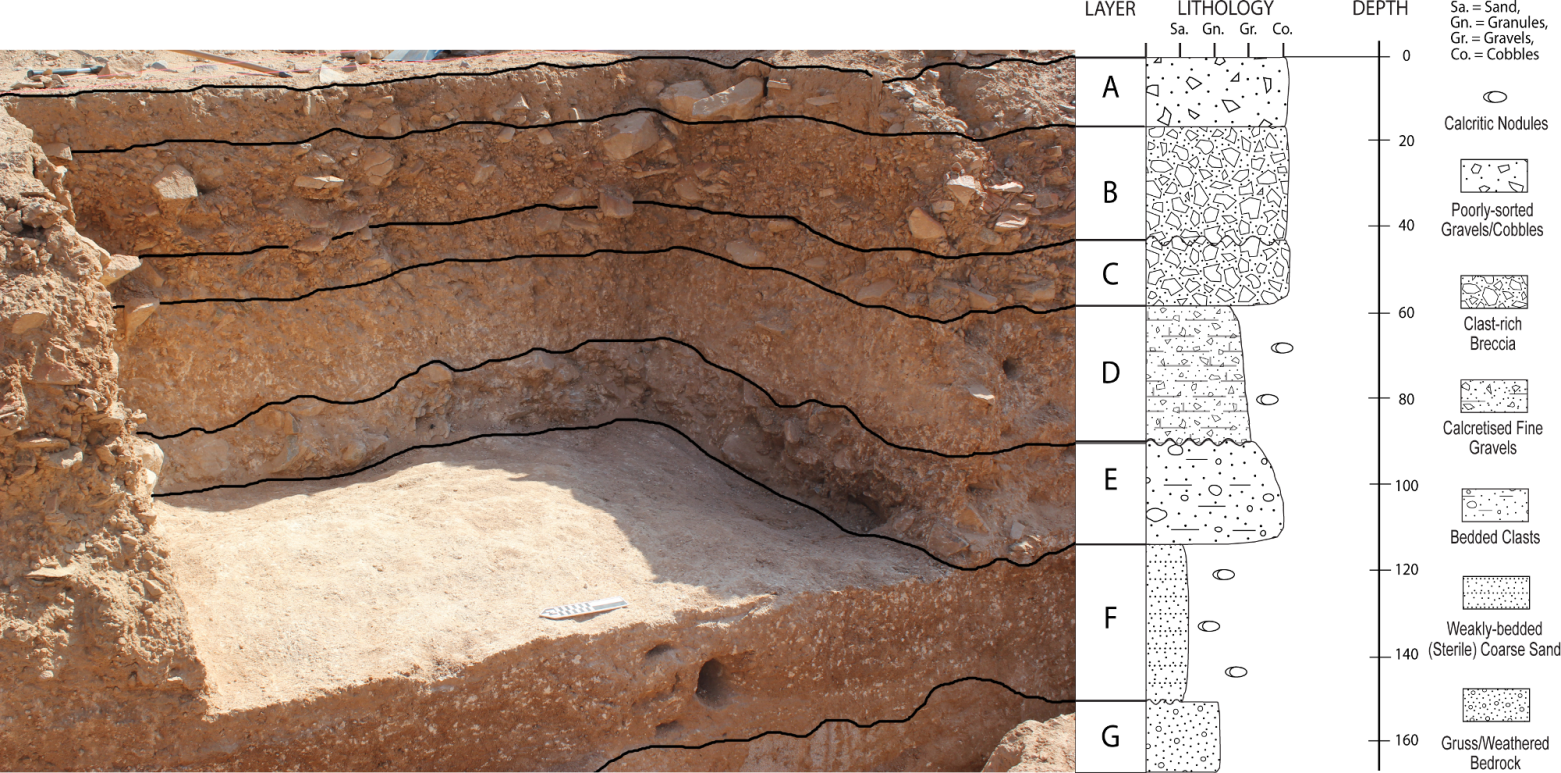
The surface of Layer F exposed, looking southeast, with the seven layers differentiated. Note the high frequency of clasts in a fine matrix in Layer E, the paucity of clasts in Layer D, and the clast supported matrix of Layers B and C.

Our 2014 excavation at Saffaqah exposed seven stratigraphic units (Layers A-F) visible in Fig 12, each of which we describe in turn. A basal Layer, G, was not excavated in the trench but exposed by a 50 cm wide, ~50 cm deep sondage running the 11 m length of the eastern wall of Whalen’s trench.

The stratigraphic sequence within the trench is underlain at 1.53 m by Layer G. This is comprised of granite boulders that have been chemically weathered *in situ* to form a horizon of gruss, which dips northwards downslope. No andesite clasts were observed within this stratum.

Layer F (context 11–1.70–1.15 m) was archaeologically sterile, and comprised of coarse-medium red-brown aeolian sands containing no clasts (Fig 12). It was very weakly bedded with numerous calcareous nodules and exhibited a diffuse contact with the stratum below. The absence of clasts from this level indicates the colluvial apron did not reach out as far as the trench at the time this was deposited.

Layer E (context 10–1.15–0.81 m) was the main artefact-bearing stratum and the lowest in which unweathered, natural andesite clasts were present (Fig 12). Around half of the clasts in Layer E had been flaked; and although this ratio was not quantified, this was a noticeably higher proportion than the upper strata, likely explained by a combination of relatively fewer clasts compared to the colluvial layers above and a high artefact density. Layer E was excavated in four spits with a maximum artefact density of 239.3 g/l in the second spit. The sedimentary matrix comprised a fining-upwards sequence of pale brown sandy silt, with frequent medium to large, angular clasts, and occasional small, rounded granulitic clasts. Layer E exhibited a sharp contact with Layer F and had numerous calcareous nodules throughout. The eastern side of the exposed trench face was notably more compacted and contained more natural clasts. The artefacts from Layer E were extremely fresh and both artefacts and natural clasts were horizontally-bedded. As such, it is likely that this stratum represents a primary context clast-littered surface that was extensively exploited by hominins.

Layer D (contexts 8 and 9–0.81–0.66 m) was incipiently calcretized, being comprised of pale brown-orange sandy silts with frequent soft calcareous nodules throughout, and with occasional small angular clasts of andesite. Artefact density was lower in this stratum than any other bearing artefacts except the uppermost, Layer A (25.4 g/l in context 8 and 17.8 g/l in context 9). Layer D sloped towards the east (similar to the modern surface), with a higher frequency of clasts and greater consolidation characterising the eastern part of the trench. Occasional small natural quartz pieces were found in the NW part of the trench in this horizon. The layer marks a change in the depositional regime at the site, characterised by the influx and infilling of fine sediments.

The overlying Layer C (context 7–0.66–0.52 m) was an artefact-rich (111 g/l) breccia comprised of large, angular cobbles and boulders within a matrix of medium-fine red sands. It was heavily calcretized and laterally variable, possibly due to clast creep downslope. Many of the granite clasts in this context were friable, possibly indicating that following deposition, this had been a stable surface for some time on which the granite had been sub-aerially weathered.

**Fig 13:**
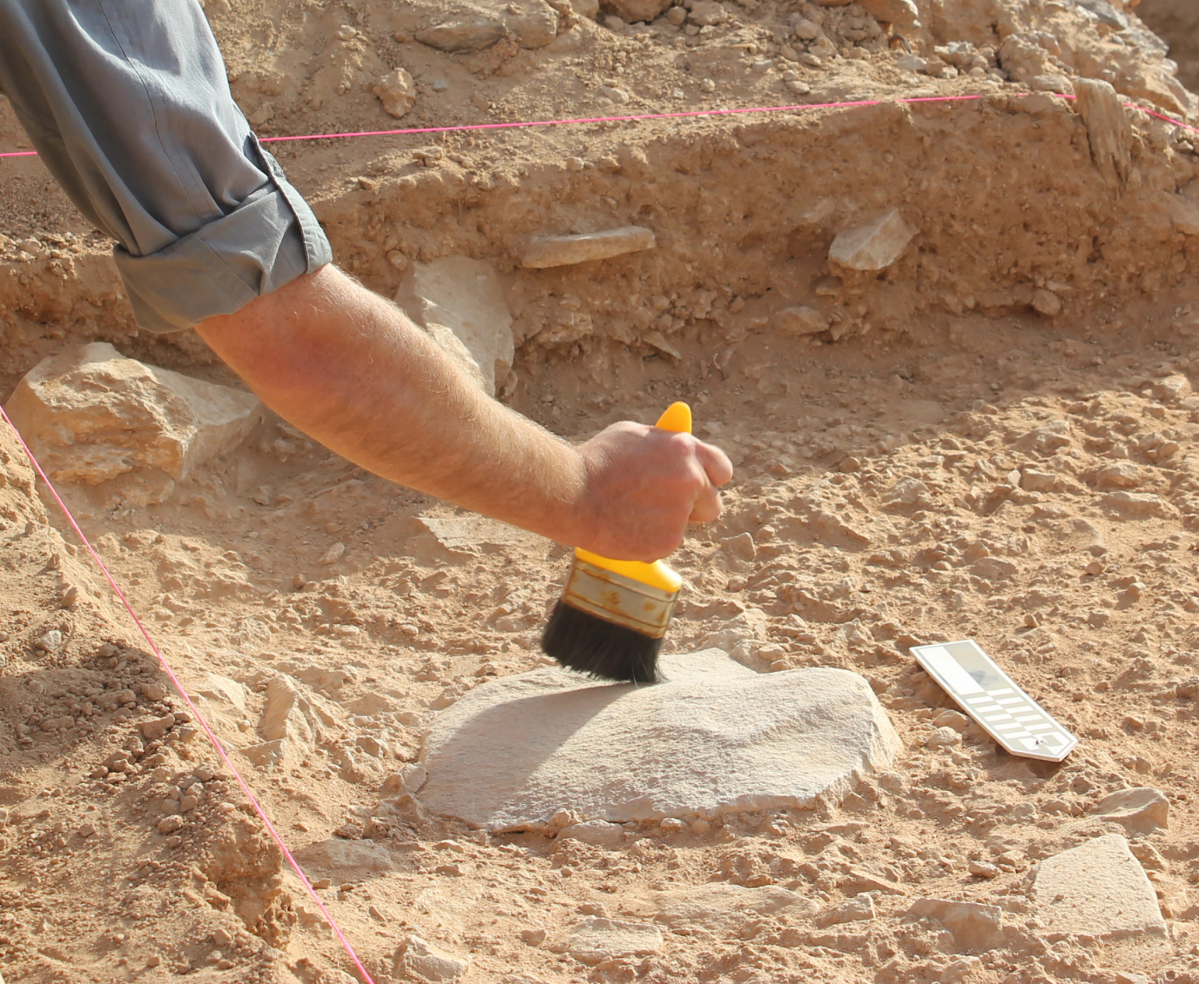
A giant flake lying horizontally on the surface of context 2.

Layer B was divided into contexts 6 to 2 (0.52–0.21 m). The interface between these contexts comprised soft, red sands and contained smaller clasts than the main context, which were cemented pale brownish-orange weakly bedded sandy-silts with gravel clasts. The larger artefacts in this layer were horizontally bedded, with the upper context (2) in this sequence having two very large flakes lying flat on the surface (Fig 13). Layer B gravels comprised small to medium angular to rounded iron-stained clasts, which have calcrete formed over the lower surfaces. This layer represents colluviation, typified by the downslope deposition of loose unconsolidated material at the base of the jebel. The large size, fresh condition, and horizontal bedding of the artefacts however, suggests that many have not moved far or are perhaps in their primary positions and just buried by colluvium. The succession of these contexts suggests multiple cycles of colluviation, with differences in artefacts between contexts confirming that they were deposited at different times. Context 3 for example, unlike any other context, contains several quartz artefacts, while artefact density fluctuates from a minimum of 35 g/l in context 3 to a maximum of 206 g/l in context 5.

Layer A (context 1) comprises a ~20 cm thick layer of orange-brown silty sands, which are moderately cemented and feature frequent small to large, poorly sorted angular clasts of granite and andesite. There were occasional medium sized lithics in this stratum, with smaller flakes appearing towards the base of this context, perhaps through post-depositional settling in the sandy matrix. The contact with the context below was diffuse and redder with fewer clasts. Layer A represents aeolian sand mixed with silt from occasional minor slopewash events. Iron staining of the upper clasts (context 2) (Fig 3) is likely due to post-depositional weathering by occasional wetting of the surface. It is possible that the surface artefacts were once buried and then have been re-exposed through aeolian deflation, but the artefacts at the site are protected by the dyke and do not appear to have suffered extensive aeolian remodelling.

**Fig 14:**
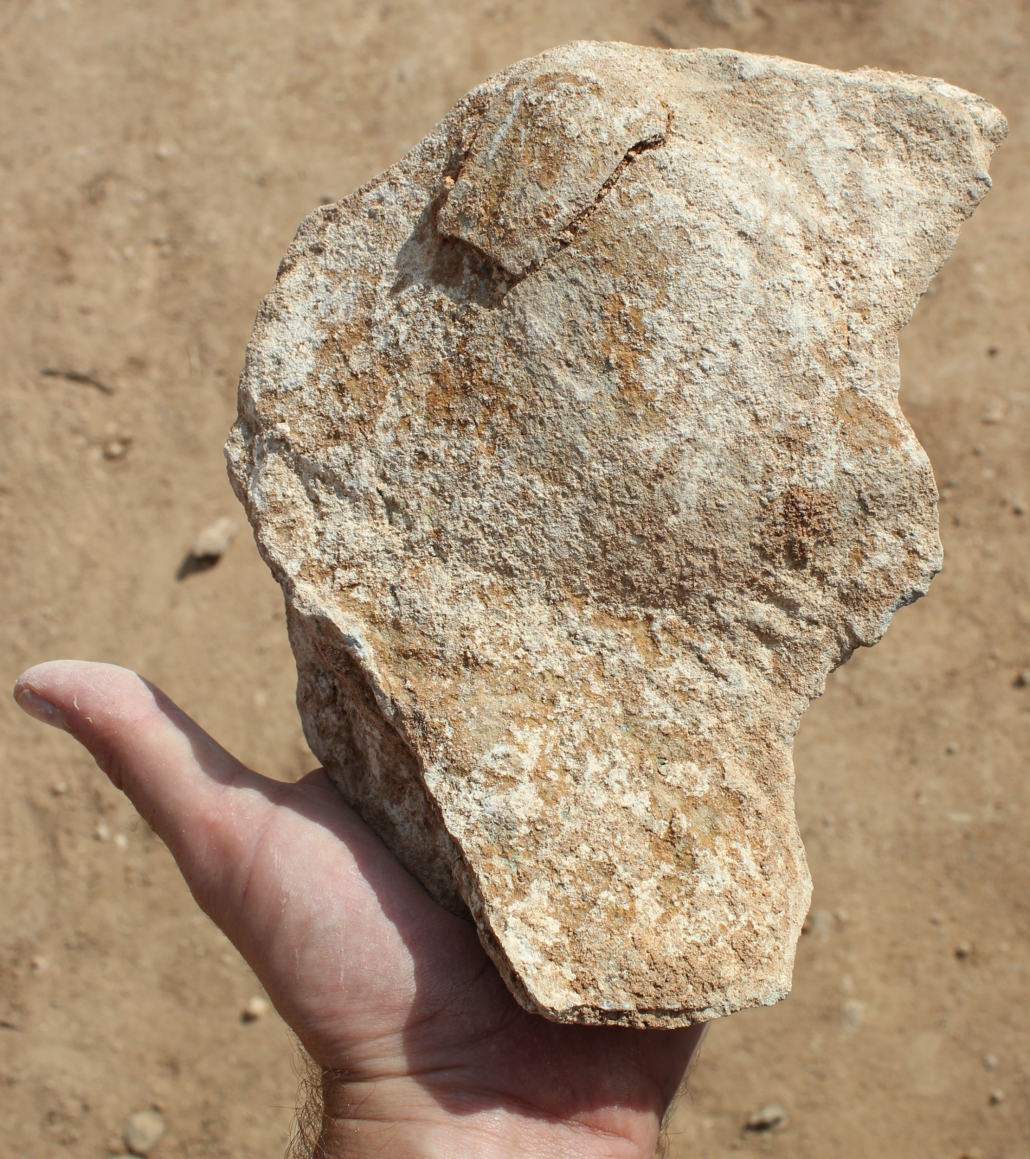
A giant flake from Layer E with the erraillure flake still adhering to it.

In comparison to those on the surface, the artefacts recovered from the excavation included ones smaller in size (<25 mm) (Fig 6), probably because the buried artefacts had been protected from erosion. The excavated artefacts were also far fresher than those on the surface with some, particularly those from Layer E, in pristine condition. Indeed, one of the large flakes from layer E was still resting on the core from which it had been struck with its eraillure flake adhering to it (Fig 14).

The lack of exploitation of the dyke itself suggests that hominins were content with the eroded clasts and occasional large blocks of andesite that could be found at the base of the slope. In the case of Layer E, where very fresh artefacts occur in a depression and are buried by a fine matrix, the artefacts are likely in the positions in which they were discarded.

Our excavation of Saffaqah 206–76 suggests the following depositional scenario took place over what was likely quite a considerable length of time, allowing for the accumulation of over 1.5 m of sediment in seven different layers. The basement granite rock weathered into boulders that were degrading in situ (Layer G). On top of these, fine silty sediment was deposited in a depression in unit T of Whalen et al.’s trench (Layer F), likely during a period of increased humidity when water mobilized fine sediment. As the colluvial apron of the dyke expanded with its ongoing erosion, andesite clasts were deposited at the trench locality (Layer E). Hominins arrived at the site when Layer E was exposed and extensively exploited the andesite, as well as introducing and working occasional clasts of rhyolite. This was followed by low energy deposition burying the initial occupation layer in finer sediment though still with some colluvial input (Layer D). Given the diffuse contact between Layers E and D, we suggest that the strata represent quasi-continuous deposition/occupation at the site. The final three Layers (C-A) represent a colluvial-aeolian sequence with cycles of stability and increased deposition rates, with artefacts throughout indicating hominins were still occupying the site during this time. We suggest that the environmental change triggering the erosion of the dyke and the colluvial accumulation of andesite clasts at its base was increased rainfall. This seems to have stopped at around the same time as the Acheulean occupation ended, as there are no artefact-free layers of colluvium overlying the archaeological sequence.

### Artefact distribution in the excavation of Whalen and colleagues

The distribution of sediment deposits and artefacts through the sequence offers some insight into depositional processes at the site and diachronic patterns in andesite exploitation. A histogram of length was plotted for all excavated flakes to assess overall artefact size distribution (Fig 6). The small size of some excavated artefacts coupled with the unimodal left-skewed (right-tailed) size distribution (Fig 6) attests to the high integrity of the excavated assemblage [33]. This accords with the small size and pristine condition of some of the artefacts from our own excavation.

**Fig 15:**
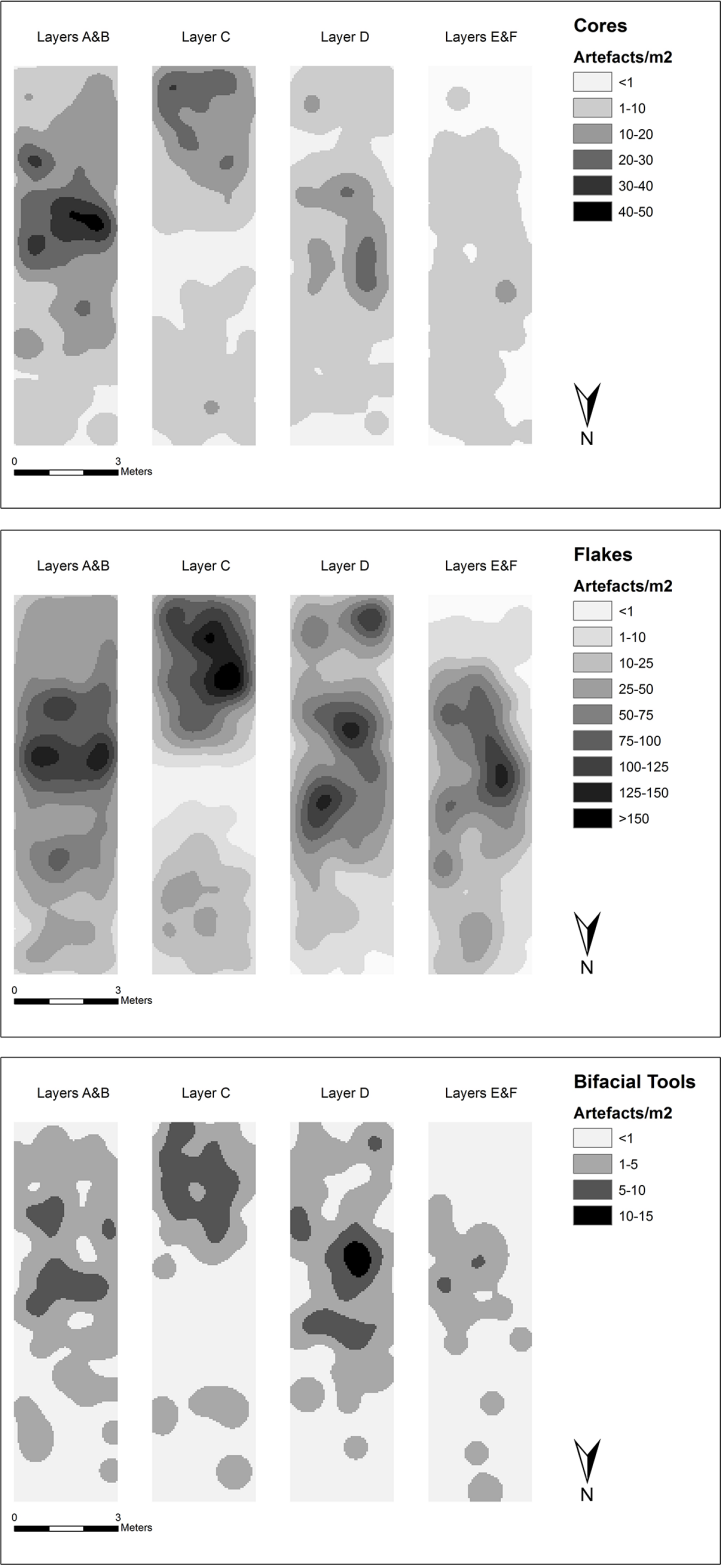
The horizontal distribution of cores, flakes, and bifacial tools in the Whalen Trench divided by layers.

**Fig 16:**
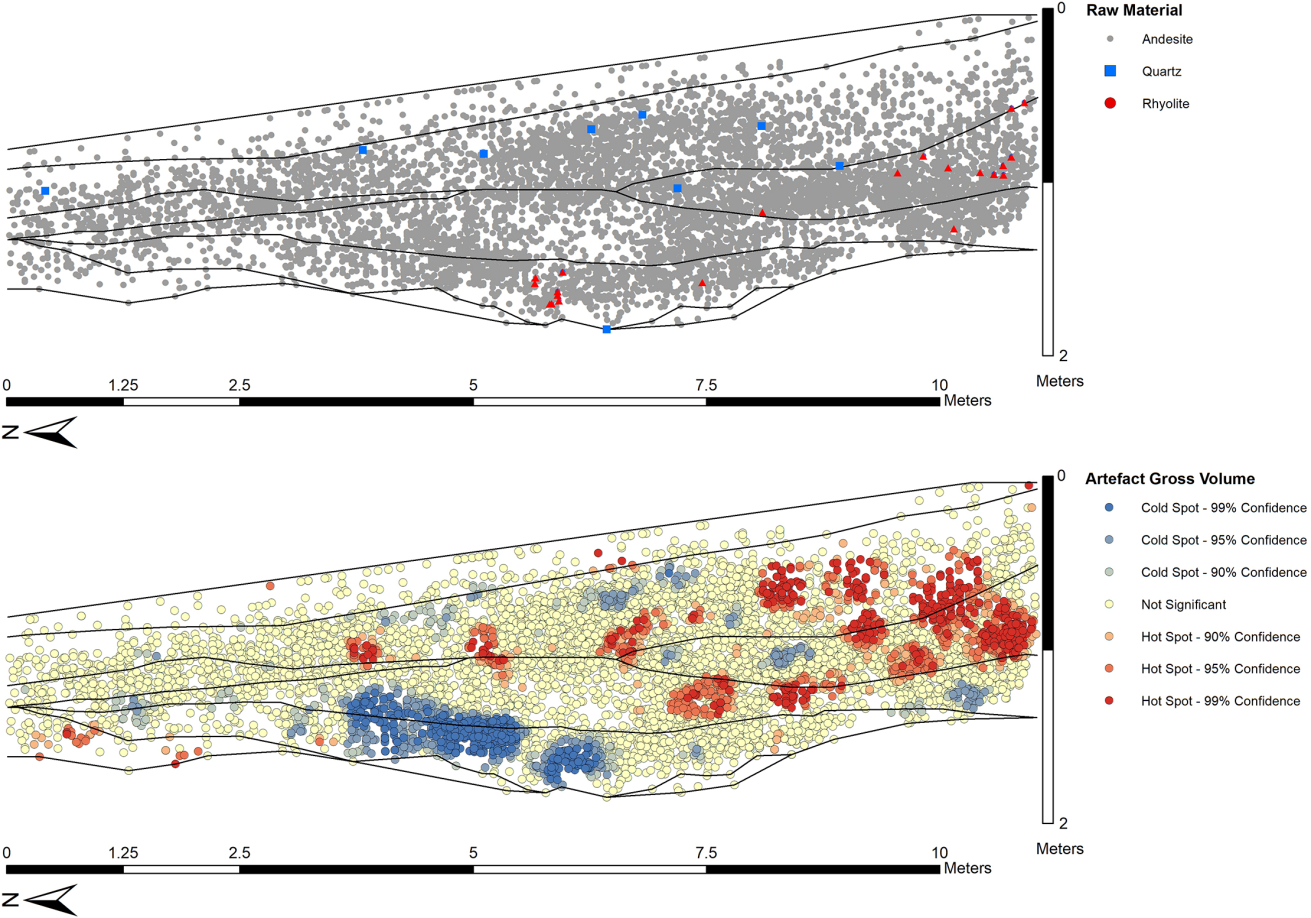
The vertical distribution of artefacts in the Whalen Trench by material, size, and density. Layers were drawn by the present authors and correspond to those in the 2014 excavation. Only the upper part of Layer F containing artefacts is shown. Note that the vertical scale is exaggerated.

Artefact densities within each Layer (A/B, C, D, E/F) were calculated and plotted for all cores, flakes, and bifacial tools (bifaces, handaxes, cleavers) to examine vertical and horizontal variability in distributions (Figs 15 and 16). The earliest cultural horizon, Layer E, occurs in a depression in the middle of the trench, and among the artefacts there is an emphasis on flakes and bifacial tools, with relatively fewer cores, and generally smaller artefacts. This locus of artefact deposition continues in Layer D, where it turns from a depression to a low hump. The main focus of deposition in Layer C is shifted upslope (south), associated with high flake and bifacial tool densities and large artefacts, with more diffuse artefact accumulation observed downslope, beyond the raised deposits of Layer D. In Layers A/B the focus of artefact deposition returns downslope to a similar position observed in Layers D and E, with high concentrations of both flakes and cores in this location and generally larger artefacts.

The overwhelming majority of artefacts are produced on andesite (99.6%), with a few artefacts made on quartz (n = 9) and rhyolite (n = 21). Quartz artefacts are predominately found in Layer B (n = 7), with single artefacts recovered in Layers C and E. In contrast, rhyolite is prominent in lower deposits, with Layers C and E each yielding 9 artefacts, with low counts in Layers D (n = 1) and B (n = 2). Of these imported materials, one of the rhyolite artefacts is a cleaver, a further six rhyolite and two quartz pieces are retouched and one quartz piece is a core.

Statistical analyses were conducted as described in the Methods, with P-values and descriptive statistics reported in S1 File. Variability in artefact class composition was identified between the layers (Fig 15). Layers A/B have a high proportion of cores (18.9%), while Layers D and E/F have a significantly low proportion (11.4% and 7.8% respectively). Layers E/F have a significantly high proportion of flakes (87.5%), whereas Layers A/B have a significantly low proportion (74.7%). No significant difference in the proportions of bifacial tools was identified.

Significant variability in artefact class composition was identified between all layers (Fig 15). Layers A/B have a high proportion of cores (18.9%), while Layers D and E/F have a notably low proportion (11.4% and 7.8% respectively). Layers E/F have a high proportion of flakes (87.5%), whereas Layers A/B have a significantly low proportion (74.7%). Bifaces range between 2–5%, whereas the frequency of choppers and picks ranges between 2.5–4%.

Significant variation in artefact size occurs through the sequence (Fig 16). Among the core assemblage, items from Layers A/B are significantly smaller (mean length = 142.6 mm; mean width = 93.59 mm; and mean estimated volume = 1,012 cm^3^) than those from Layer C (mean length = 156.8 mm; mean width = 101.8 mm; mean estimated volume = 1,212 cm^3^), but no significant differences were identified with the other layers. Flakes are large (mean length > 79 mm) throughout the sequence (Fig 6) but there are some subtle differences between layers. Flakes from Layers E/F show significantly smaller lengths, thicknesses and volumes than all other levels, as well as lower widths than Layers A/B and D. Flakes from Layer D are significantly longer and thicker than those in Layers A/B, and significantly wider than those in Layer C. Flakes from Layer C are more elongate (mean length/width ratio = 1.19) than either Layer A/B (mean = 1.17) and Layer E/F (mean = 1.16). Flakes from Layer E/F (mean width/thickness ratio = 3.42) are flatter than other levels, whereas Layer A/B flakes (mean = 3.3) are flatter than those in Layer C (mean = 3.13). The only significant results for bifaces indicate estimated volume of bifacial tools from Layers A/B (mean = 816.2 cm^3^) were smaller than those for Layers C (mean = 1,038 cm^3^) and D (mean = 1,065 cm^3^).

Overall, lower artefact densities are observed toward the downslope end of the trench, where sediment horizons are thinner. Artefact deposition is concentrated in the middle of the trench where the sediment is thickest with repeated episodes of high artefact density in three of four horizons. The artefact assemblage from the lowest excavation levels (Layers E/F) is comprised of a greater proportion of smaller flakes than overlying horizons, but no significant differences in the size of cores or bifacial tools. This suggests a greater intensity of knapping activity occurred at this time with a high ratio of flakes to cores, and, as noted in the 2014 excavation, a high proportion of artefacts relative to natural clasts. Another explanation, not mutually exclusive, is that this lower stratum has undergone the least post-depositional disturbance with the small flakes not winnowed away or destroyed, as they may have been higher up the sequence. This lower horizon also contains many of the rhyolite artefacts unlike Layer D, which is notable for the artefacts being comprised almost exclusively of andesite, with only a single rhyolite artefact and no quartz present. Layer D contains both the largest flakes and the highest estimated volumes for bifacial tools, while there were relatively fewer cores. Layer C presents cores and bifacial tools that are significantly larger than those in the overlying Layers A/B. Rhyolite artefacts are relatively common in Layer C, while most of the quartz artefacts occur in the overlying Layer B. Layers A/B have the highest proportion of cores and bifacial tools relative to flakes, which are both typically smaller than those in the layers below. One possible explanation of this pattern is that dyke erosion was very active during the deposition of Layers A/B with successive waves of colluvial deposition resulting in an abundance of andesite clasts. These clasts could then have been used profligately with many smaller cores yielding only a few flakes before being abandoned, and smaller clasts of appropriate shape more often made into bifaces. Overall, the distinctions in artefact types and materials between the layers attests to the integrity of the trench in general and the lower layers in particular.

### Technology

#### Survey

The Acheulean assemblages at all of the major Saffaqah localities are either dominated or exclusively represented by igneous stone artefacts, with large flakes (>100 mm) and cleavers being universal features (Table 1). Technologically the assemblages therefore demonstrate a considerable degree of homogeneity.

**Fig 17:**
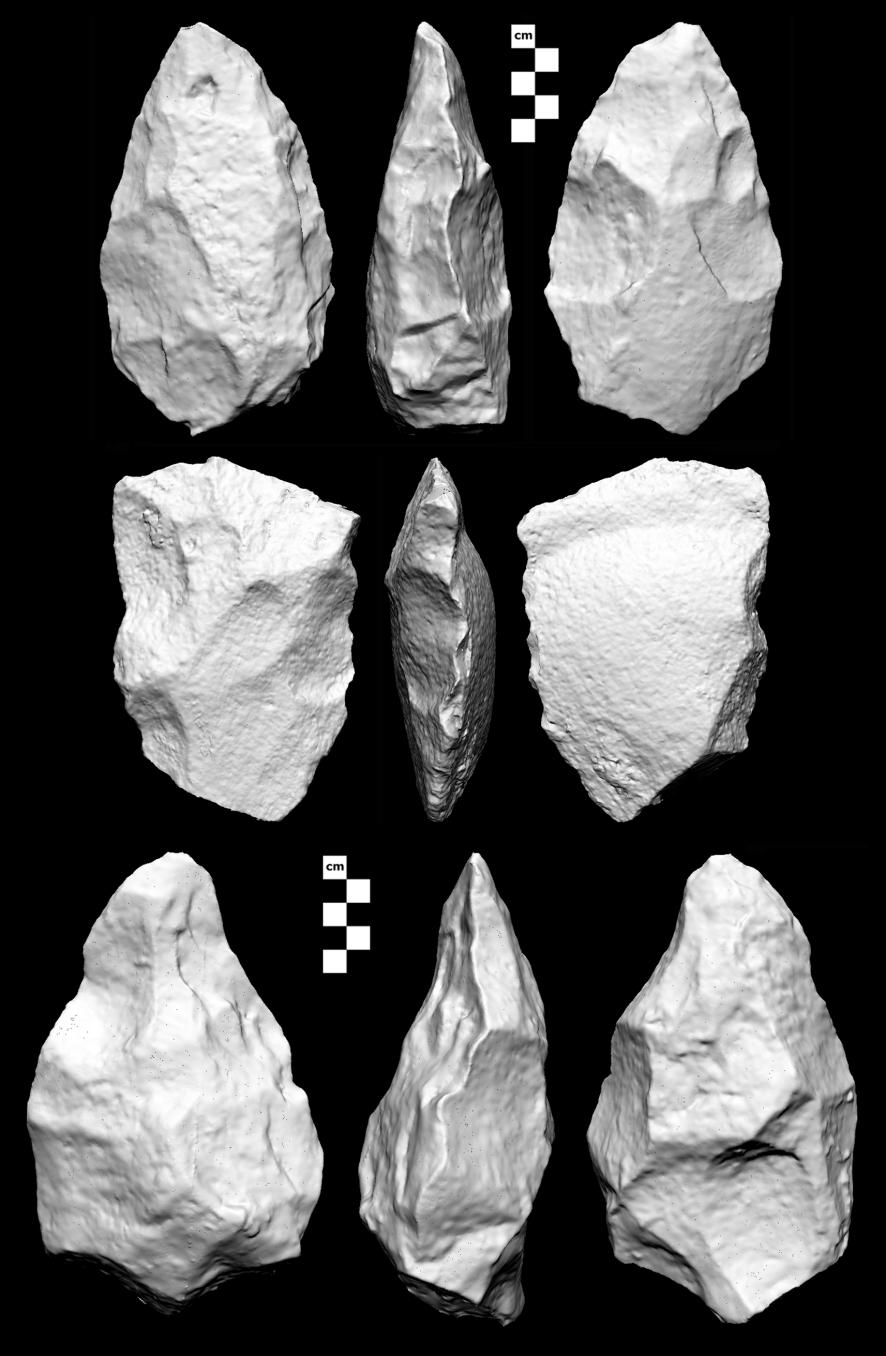
**Handaxe (top), cleaver (middle), and early stage biface (bottom) from Saffaqah.** The handaxe and cleaver are made on large flakes, while the early stage biface is made on a cobble. The handaxe is from Saffaqah 177 and is 165 mm long. The cleaver is from Saffaqah 206–76 surface and is 144 mm long. The early stage biface is from Saffaqah 206–76 excavation context 10b (Layer E) and is 193mm long.

Where blank type was discernible, 80% (N = 105) of the Saffaqah bifaces encountered during the survey were made on flakes (Fig 17). This high proportion of flake-made bifaces is likely an overestimation, as some bifaces made on the larger cobbles that are available at Saffaqah will have lost all trace of their original blank by the time they were shaped into handaxes. Cleavers were only a minor component of the biface assemblage (3.3%, N = 19), but were represented at most of the main sites including 206–76 (Fig 17). The mean of biface refinement (thickness/width) for all bifaces recovered during the survey was 0.579±0.135 (N = 341), which is toward the thicker end of the Acheulean range [34].

**Fig 18:**
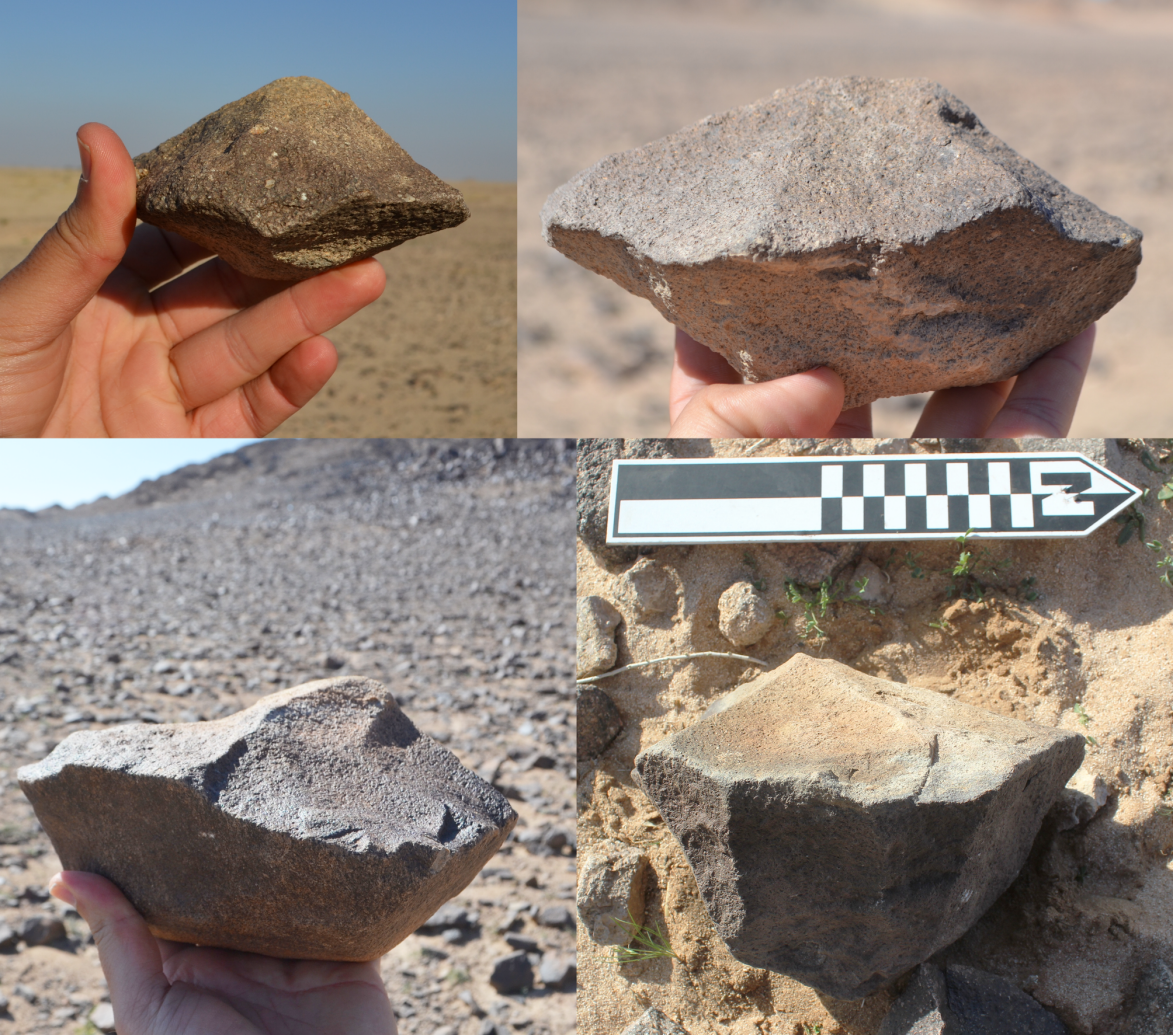
Four discoidal cores from Saffaqah. Note that the core on the bottom left has an unequal plane of intersection and would be classed as hierarchcial, but that its upper surface is still conical like the other discoidal cores.

Large flakes for biface blanks were produced through the bifacial flaking of appropriate edges on large blocks of andesite as evident at 206–76 (Fig 10). Similarly for smaller cores, 82% (N = 39) of those assigned to type during the survey were some variety of bifacial core, be they semi-discoidal (discoidal cores that were not flaked around the entire perimeter), discoidal, or hierarchical discoidal cores (discoidal with a pronounced asymmetry in the intersection height between the upper and lower surfaces) (Fig 18). The remaining cores were single and multi-platform.

**Fig 19:**
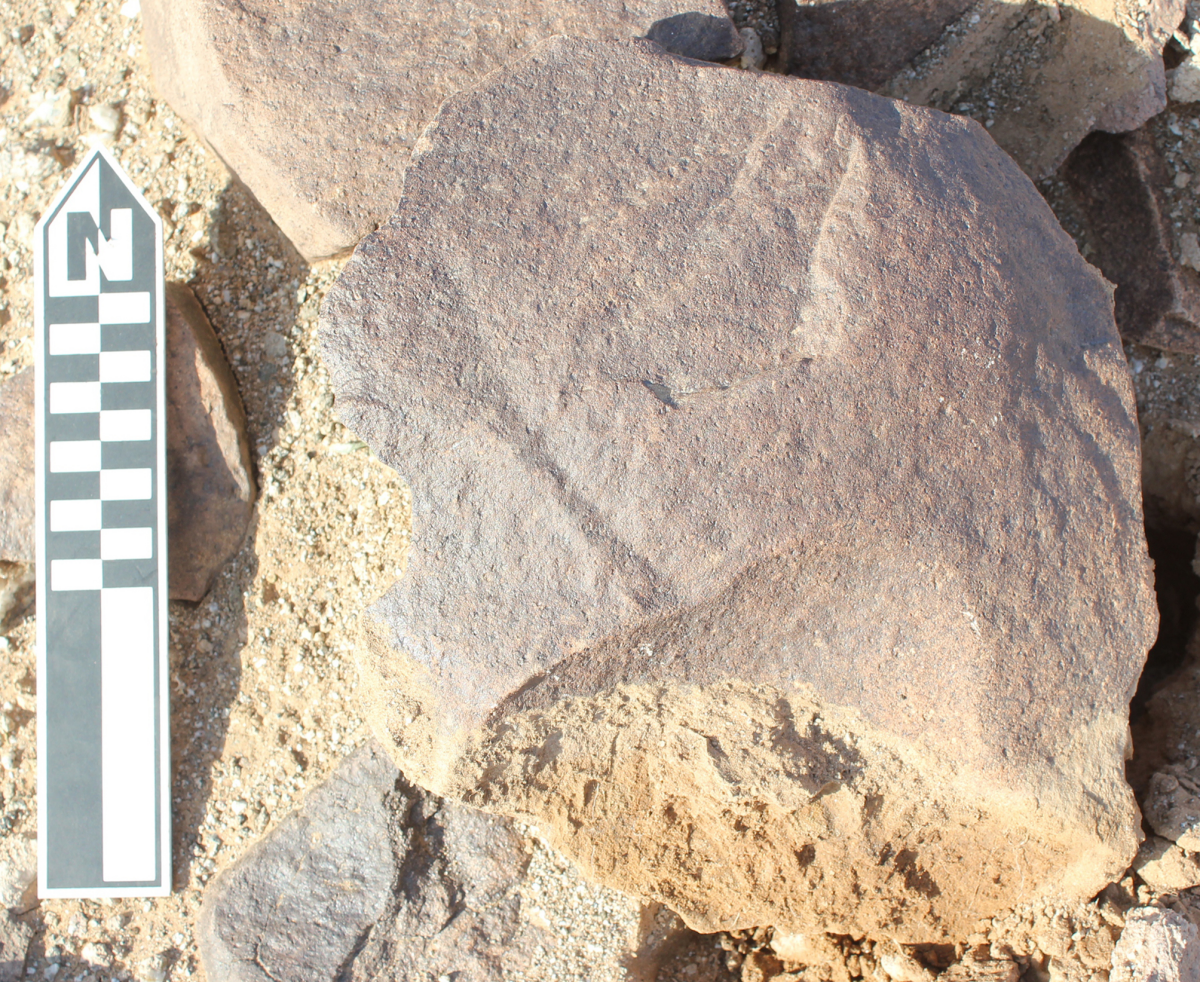
A giant flake with a single large scar on the ventral found during the Saffaqah surface survey.

The pattern of retouch on the Saffaqah flakes was unusual, with 86.7% (N = 30) of cases in which retouch location was recorded, being on the ventral surface only (Fig 19). Mean length for the ventrally retouched flakes was 118 mm, with 23 out of 25 specimens being larger than the shortest flake-blank biface (80 mm). It is therefore than many of these ventrally retouched flakes were biface preforms discarded at an early stage of reduction, with the ventral retouch done in order to move the plane of intersection between the flatter ventral surface and the more domed dorsal surface into a more central position, thereby creating a pseudo-bifacial edge.

#### Saffaqah 207–76 surface

**Fig 20:**
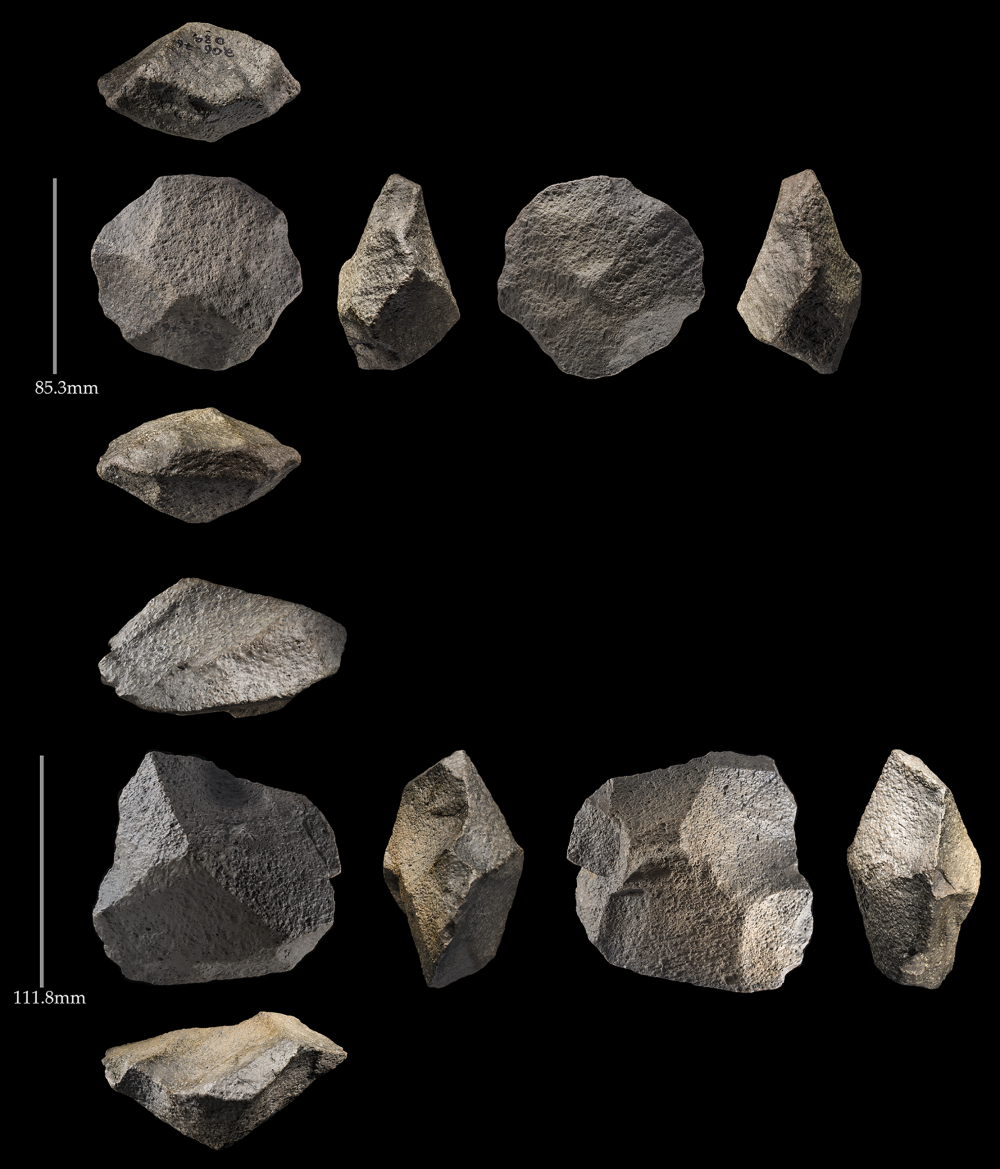
Examples of discoidal cores from Saffaqah 206–76. Note that both surfaces are centripetally worked with scars terminating at peaks in the centre of the pieces.

This sample of 50 cores were mostly discoidal, semi-discoidal, or hierarchical discoidal, with only 2 being single platform (Fig 20). Two of the hierarchical discoidal cores had conspicuously larger scars on the upper surface similar to Levallois cores, and indeed Whalen et al. [27] reported several Levallois cores at the site. However, there was no sign of convexity preparation on the upper surface of these cores, with scars converging at a central peak, so we regard these as fortuitous rather than a deliberate use of the Levallois technique.

Non-feather terminations were apparent on only 15% of scars from the 50 cores (N = 709), so the Saffaqah hominins were usually able to strike with enough force that there was no need for the energy to exit early and abruptly from the core. Furthermore 32% of scars travelled at least 1/3 of the distance across the face of the core, indicating these hominins had no difficulty in striking large flakes.

Forty-two (84%) of the cores have scars 80 mm or larger in maximum dimension that could have been from biface blank flakes; while 29 (58%) have scars longer than 100 mm corresponding to other definitions of large flake technology [35]. The largest flake scar measured 250 x 200 mm. Nine of the cores were large enough to be considered giant (greater than 250 x 200 mm in maximum dimensions) [36].

Around half (N = 24) of the dominant flake scars on each core were longer than they were wide (end-struck), while the other half (N = 26) were shorter in length than width (side-struck), the mean size of these scars being 101.3±37.9 mm long, and 97.5±36.5 mm wide. This compares with mean dimensions for flake blank handaxes recovered on the survey of 129.9±21.8 mm long, and 76.7±13.34 mm wide (N = 52), while flake cleavers were 132±19.9 mm long, and 85.3±10.86 mm wide (N = 7). The greater elongation and standardisation in length and width of the bifaces, as opposed to the large flake scars, likely reflects the imposition of biface form through retouch.

#### The 2014 excavation

The cores from the 2014 excavation include an assayed cobble (a core with less than four scars), a single platform core, a discoidal core, and a hierarchical discoidal core. In keeping with the discoidal nature of the Saffaqah technology most platforms on flakes from the excavation were either formed from a single scar or were dihedral (56.6% and 16.4%, N = 152).

**Fig 21:**
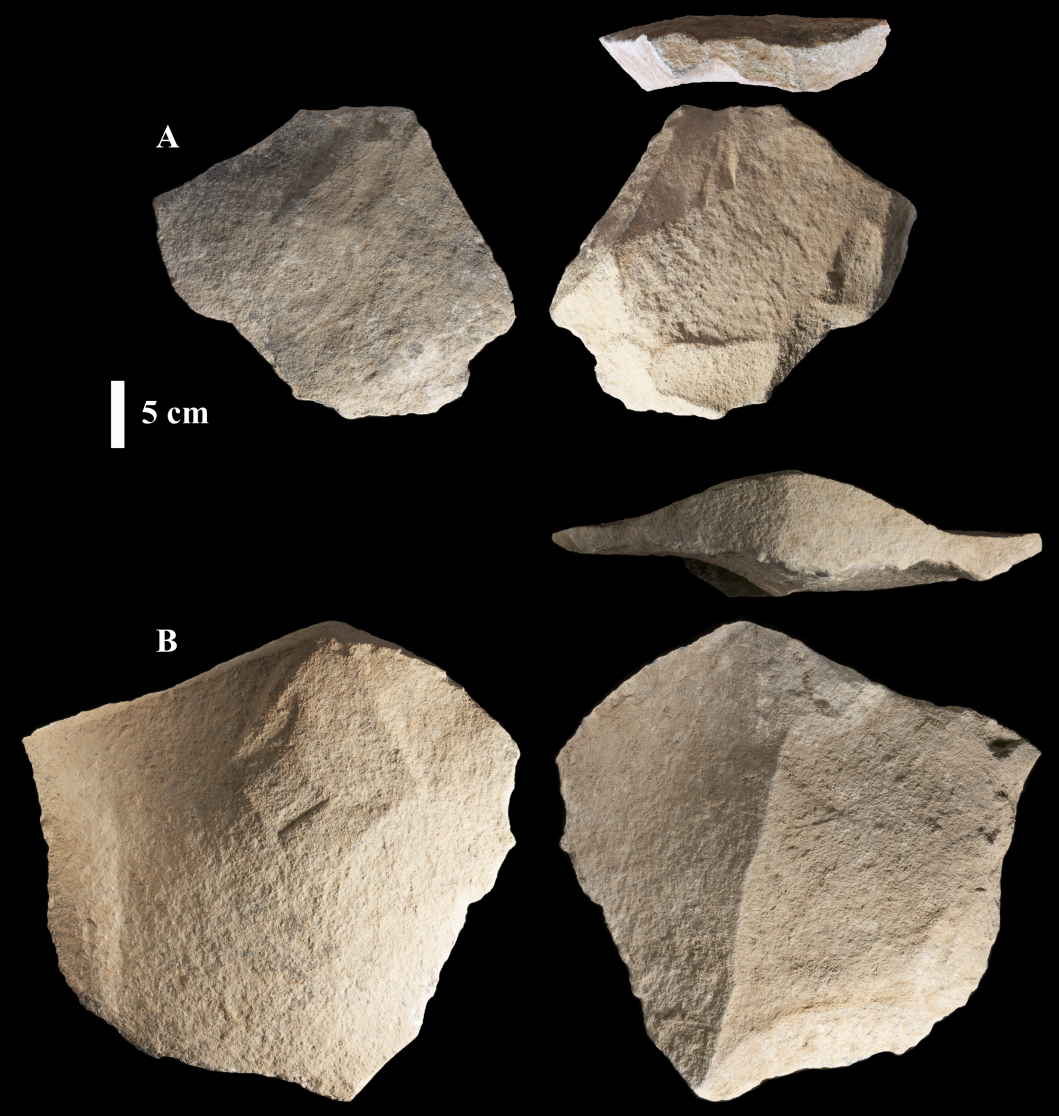
Giant flakes from the Saffaqah 206–76 excavation, Layer B. Note the shape of the platform on specimen A, created by two cones of percussion, the groove separating them can be seen on the ventral surface.

The excavated material captures the lower end of the size range with flakes as small as 13 mm in length. For the surface survey the smallest flakes recorded were only 25 mm long, since sandblasting of artefacts lying on the surface had destroyed the smallest flakes, or at least obscured their identification as artefacts (Fig 6). At the upper end of the size range the surface survey included flakes up to 390 mm long, and in the excavation the two largest flakes were 240 mm and 310 mm in maximum dimension (in the size range of the largest scar recorded on the cores) (Figs 19 and 21). To be able to strike such large flakes clearly requires great strength. Only 9.9% (N = 162) of flakes had hinge or step terminations, while there were two instances of overshot flakes testifying to the strength of these knappers. This compares with, for example, 24% of flakes (>20 mm) from the Middle Palaeolithic site of Jebel Katefeh 1 (JKF1) in northern Arabia having hinge or step terminations [37]. A high proportion of longitudinal siret breaks (11.8%, N = 187) also might be due to excessive force. A hammerstone was found from context 2, which has a maximum dimension of 115 mm and weighs 990 g. Whalen found nine hammerstones ranging from 178 to 84 mm in length. Such hammerstones could have been used to create some of the larger flakes. One of these large flakes has a double cone of percussion (Fig 21A). This may be because it was struck from a flat platform, such as occurs on some of the larger *in situ* blocks of andesite (Fig 10), by a relatively flat hammerstone; thus there were two points of impact. Alternatively, it might be because the initial attempt to detach the flake was unsuccessful but left an incipient cone and then the flake was struck from the same platform.

Two early stage bifaces were recovered from context 3, one of which was on a flake blank. Fig 17 shows another andesite biface from context 10b, likely made on a clast of andesite given its thickness, and showing an attempt to reduce that thickness through a scar struck from the butt of the piece. As it is a complete scar the biface may have been abandoned after this failed attempt. In general biface flaking was not very invasive which contributed to the failure to thin the piece here, while bifaces made on smaller blanks often retained cortex and/or dorsal scars in the case of flake blanks (Fig 17). The lack of thinning on these excavated bifaces is in accordance with the low refinement values of the larger sample of bifaces from the survey.

Inspection of the artefacts from the excavation could not identify any notable differences in technology across the sequence with the production of large flakes and bifacial flaking a persistent feature throughout. To quantitatively test whether there was a diachronic trend in knapping technology at the site, flakes from contexts 1–3 (Layer A and the upper part of Layer B), were compared with those from context 10a (the upper part of Layer E). Platform type selection is an important variable in knapped stone technology, affecting the dimensions of the resultant flake [38], and varying according to different Acheulean biface knapping strategies [39, 40]. The proportion of single, dihedral, and multiple scar platforms were compared between the two samples using a chi-squared test (Table 2). The test indicated that there was not a significant difference (P = 0.683) in hominin platform selection between the upper and lower parts of the sequence. Platform thickness is another important knapped stone variable affecting the size of the flake produced [41] and varying according to stages of Acheulean biface reduction [42]. A t-test showed that there was no significant difference between flake length to platform thickness ratio between contexts 1–3 and context 10a, (contexts 1–3 mean = 5.084, SD = 2.923, N = 58; context 10a mean = 5.1972, SD = 3.72, N = 103; P = 0.793). Both the surface and excavated artefacts point towards constancy in the character of the Saffaqah Acheulean.

**Table 2:** The proportion of different platform types for flakes in contexts 1–3 (Layer A and the upper part of Layer B) and context 10a (the upper part of Layer E).

	Single	Dihedral	Multiple	Total
**Contexts 1–3**	30 (64%)	12 (25%)	5 (11%)	47 (100%)
**Context 10a**	56 (70%)	17 (21%)	7 (9%)	80 (100%)

## Discussion

Saffaqah 206–76 is still the only stratified Acheulean locality yet to be documented in Arabia. Our findings demonstrate the presence of a population of Acheulean hominins using bifaces produced on large flakes and cobbles, as well as discoidal cores for the production of small flakes. Such a dual pattern of large shaped bifaces and smaller flakes struck from portable cores is typical of the Acheulean [43–45]. Saffaqah 206–76 is the largest locality of the Acheulean period in Arabia and occurs in the midst of an extremely dense Acheulean landscape. The presence of terracettes, the differential patination on the artefacts, and the small size of the wadis in their bedrock-constrained courses all point towards the stability of the landscape. Furthermore, the underside of some excavated artefacts being covered in calcrete, the distinctive typological and material character of the different layers of the excavation, the horizontal bedding of the artefacts and their freshness, and the size distribution of the flakes all point towards the integrity of the site itself.

Elsewhere in the Arabian Peninsula Acheulean hominins were creating large flake blanks on igneous rock and making cleavers in a number of locations including Wadi Fatima on the Red Sea coast [46], and in the southern Nefud Desert [8] (Fig 1). In the Levant, at Gesher Benot Ya’aqov and its sister Northern Bridge locality (Fig 1), a distinctive series of Acheulean assemblages similar to Dawadmi are characterized by the production of handaxes and cleavers through the marginal retouch of large flakes, usually of igneous rock [47, 48]. This facies of the Levantine Acheulean has been suggested to represent a dispersal from Africa during a humid period [35, 49, 50]. The Acheulean at Dawadmi likely also represents occupation during a humid period when the river systems of Arabia were also activated allowing hominins to penetrate into the interior [28]. A distinction between the Acheulean of Saffaqah and that of Gesher Benot Ya’aqov is the relative paucity of cleavers in the former. If, as has been suggested, cleavers were wood-working tools [51–53], then this difference might be explained by a relative scarcity of trees in central Arabia in comparison to the Levant.

Whalen and colleagues reported a Levallois element in the Saffaqah technology, however our analysis of the Saffaqah material did not uphold this observation. Although there are occasional cores with relatively large scars and hierarchical asymmetry between the surfaces, there are no examples of faceting or shaping the upper surface. The asymmetry in the intersection height on some cores appears to form a continuum with more symmetrical pieces rather than a different core type, with scars meeting at a central peak even on the upper surfaces of these cores. Bifaces are a numerically dominant component of the technology here, as opposed to the more minor role they might be expected to play if the assemblage were transitional [19, 53]. Artefacts in general are much larger than those of the Middle Palaeolithic. The Middle Palaeolithic artefacts that do occur in the region are extremely sparsely spread, and do not appear related to the rich Acheulean landscape [24].

The stratigraphic sequence at Saffaqah 206–76, comprising five sedimentologically distinct layers (A-E) of hominin occupation, provides the opportunity to assess diachronic change in technology in the face of environmental variation. While quartz and rhyolite were very occasionally introduced, the local andesite always accounted for the vast majority of artefacts. And while there was subtle variation in artefact size, perhaps as a function of clast abundance, there was a striking homogeneity in the technology. Large flake production was a consistent feature throughout the occupation of the site (Figs 14 and 21). Lithics from the upper and lower parts of our excavation showed no differences in either platform type proportions, or flake length to platform thickness ratios. For the larger sample of the Whalen excavation there were no significant differences in flake elongation or biface refinement throughout the sequence. The proportion of bifaces also did not significantly vary throughout the sequence. In all aspects of knapping at Saffaqah, bifacial flaking was the dominant mode, whether it be the giant cores from which biface flake blanks were produced; the shaping of bifaces; or the smaller discoidal cores. The stability of technology through the Saffaqah sequence and the pervasiveness of bifaciality is consistent with the Acheulean conservatism documented at Gesher Benot Ya’aqov [47].

That both flakes and large cobbles were used as handaxe blanks is consistent with biface reduction sequences elsewhere in the Acheulean world [36, 54–57], and speaks to flexibility on the part of Acheulean hominins in adapting their reduction process to the most appropriate available clasts. Furthermore it suggests that the biface and the means of making it were conceptually independent in the minds of Acheulean knappers [57], with the biface having significance as an object beyond any one particular immediate task. Instead, bifaces were apparently intended for multiple unknown future uses and as such were sometimes curated by hominins.

As has been documented in other parts of the Acheulean world, there was at Saffaqah limited fragmentation of reduction sequences, with a structured use of the landscape [11, 40, 58–62]. Unsurprisingly early reduction took place at the dyke workshop sites. Bifaces were then preferentially transported around the landscape, turning up at disproportionately high frequencies in both wadi sites and off-sites. Those bifaces transported away were more reduced than many of those left at the dykes, likely because the latter specimens were rejected or unfinished. The high proportion of bifaces at off-sites suggests they were often used with spatially variable resources that did not result in long-term patterning, such as butchery at isolated kill localities. Their accumulation at wadi sites may be the product of repeated kills at wadis, or hominins’ preference for occupying these localities themselves, or some combination of the two.

Notwithstanding specific functions for handaxes and cleavers, one of the advantages of the bifacial edge is the long time it takes to blunt [63, 64], so hominins seem to have been interested in transporting a durable edge. Durable edges would have negated the need to transport heavy hammerstones, which were extremely rare at the Saffaqah localities. Only two hammerstones were encountered during our intensive survey, at 206–68. Likewise, of the thousands of artefacts that Whalen and colleagues excavated at 206–76, only 9 were classified as hammerstones.

Landscape-use at Saffaqah is typical of the Acheulean in showing a preference for spatially fixed water and stone resources in the landscape and avoiding higher ground, e.g. [9, 21, 65]. The lack of artefacts crossing the dykes, even when they are very dense just below, is striking and does not appear to be post-depositional given the lack of evidence for primary flaking of the dyke (even though large boulders that had fallen down to the base of the slope were exploited). Walking or running up hills dramatically increases energy expenditure [66], while coming back down greatly increases the risk of injury [67]. Hominins were using a least-effort strategy for raw material procurement using the smaller eroded clasts that existed at the base of the jebel rather than climbing up to access the primary clasts of dyke itself. That we could not replicate Whalen et al.’s finding of artefacts on the terraces on the dyke, further suggests that hominin use of higher ground was at best fleeting. Rather, proximity to fresh water, seems to have been a key factor in determining where on the landscape the Saffaqah hominins were to be found, with very few artefacts on the southern side of the dyke where the wadis are more ephemeral (Fig 4). Such a pattern is probably partly related to the thermoregulatory adaptation of copious sweating in *Homo* and the consequent need for regular drinking [68, 69].

One of the most arresting aspects of the technology at Saffaqah was the size of some of the flakes and flake scars. The low levels of aberrant terminations in both the flakes from the excavation and the scars on the Saffaqah cores, indicates hominins normally struck flakes with sufficient force that there was no need for the fracture to exit prematurely from the body of the core. To produce such large flakes shows that the Saffaqah hominins had high levels of strength and dexterity, with an unrestrained wrist like our own species and the capacity to exert high manual pressure [70, 71].

### Conclusions

Acheulean occupation extended to the heart of the now hyper-arid Arabian Peninsula. Saffaqah is the largest Acheulean site thus far documented in Arabia and is surrounded by a dense Acheulean landscape. The combination of excavation and survey allows a picture of hominin lifeways at Saffaqah to be reconstructed. Bifaces were a dominant focus for tool production: they were transported away from workshops, used at various locations out on the landscape, and often curated to streamside aggregation localities. A consistent preference for bifaces may in part reflect the durability of their cutting edges, allowing for a single ergonomic tool to be curated by foraging hominins. Repeated use of streamside locations and the strength of the knappers suggests that these hominins were not eking out their existence on the margins, but were an ecologically dominant species [13].

The preference of Saffaqah hominins for streams and stone sources and the overlap of both these resources at Saffaqah may explain why it was so densely occupied. The distribution of water and stone sources would also have constrained hominin dispersal routes across Arabia. Saffaqah sits right at the drainage divide between the Wadi al Batin and the Wadi Sabha palaeodrainage systems, and hominins may have been able to utilize the activation of these rivers as corridors to reach the centre of the peninsula from the outlets in the Gulf (*sensu* Breeze et al. [72]). Dykes with stone suitable for knapping and associated Acheulean artefacts are found for tens of kilometres to the north, west, and south of Saffaqah [25]. The use of large flake blanks and the creation of cleavers allies the Saffaqah Acheulean with that documented elsewhere in Arabia, including the Red Sea coast [4, 46], and the southern Nefud Desert around the Jubbah Oasis [8].

Hominins once penetrated into the centre of the now hyper-arid region of the Arabian Peninsula, perhaps by following major wadis upstream in conditions significantly more humid than today. The density of sites around Saffaqah suggests that they flourished here. There is a consistency to Acheulean behaviour at Saffaqah that resonates with the Acheulean elsewhere in the world: hominins preferentially curated bifaces and they favoured fixed and easy to access stone and water sources. The Saffaqah hominins were technologically conservative. The lack of transitional features in their technology and the low density of Middle Palaeolithic artefacts in the region, suggests eventual abandonment by this Acheulean population, perhaps with the onset of a more severe arid phase to which they could not adapt.

## Supporting information

S1 File Statistical tests of Whalen excavation artefacts. (XLSX)
