# Correction: Acheulean technology and landscape use at Dawadmi, central Arabia

**DOI:** 10.1371/journal.pone.0203488

**Published:** 2018-09-07

**Authors:** Ceri Shipton, James Blinkhorn, Paul S. Breeze, Patrick Cuthbertson, Nick Drake, Huw S. Groucutt, Richard P. Jennings, Ash Parton, Eleanor M. L. Scerri, Abdullah Alsharekh, Michael D. Petraglia

Figs 5, 7, 8, 9, 10 and 11 appear out of order and are mismatched with their captions. Please see the correct figures and captions here.

**Fig 5:**
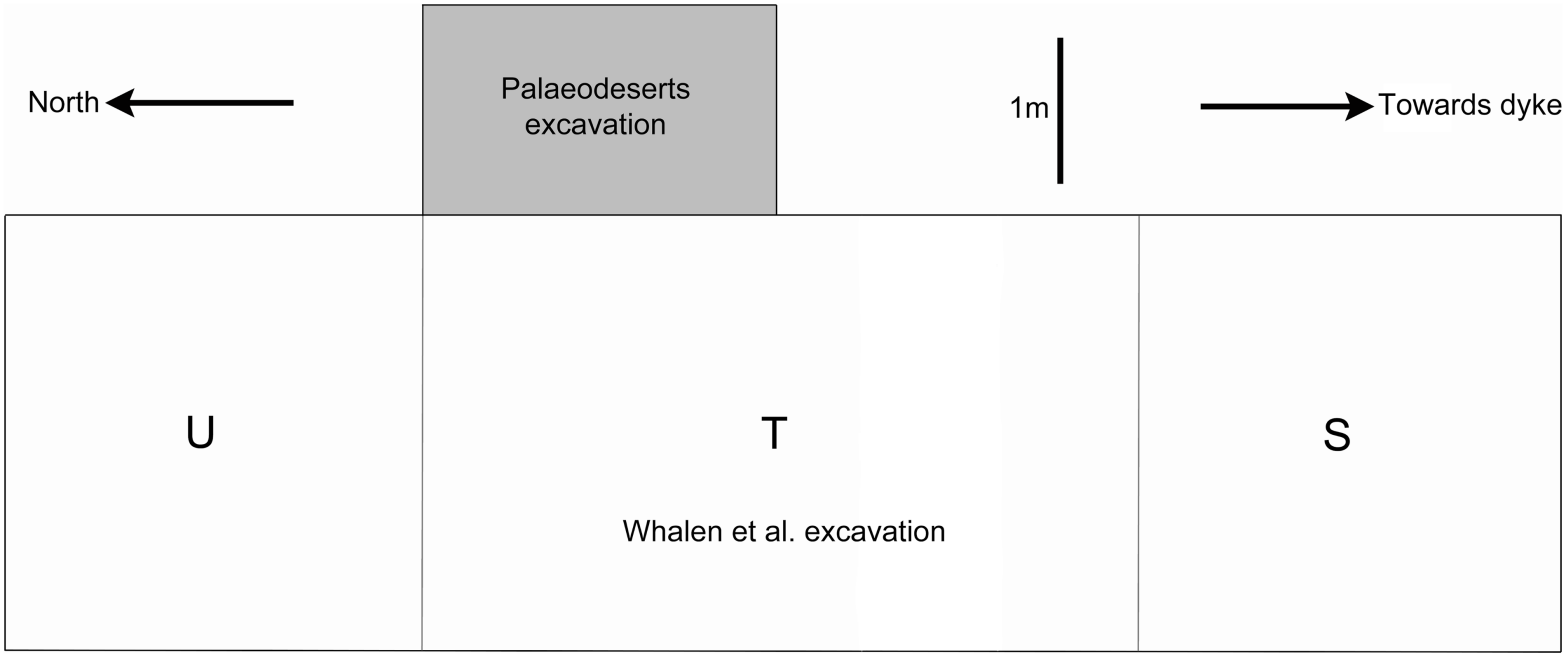
Plan of the Whalen et al. and Palaeodeserts trenches, showing Whalen’s three excavation units (S, T, and U).

**Fig 7:**
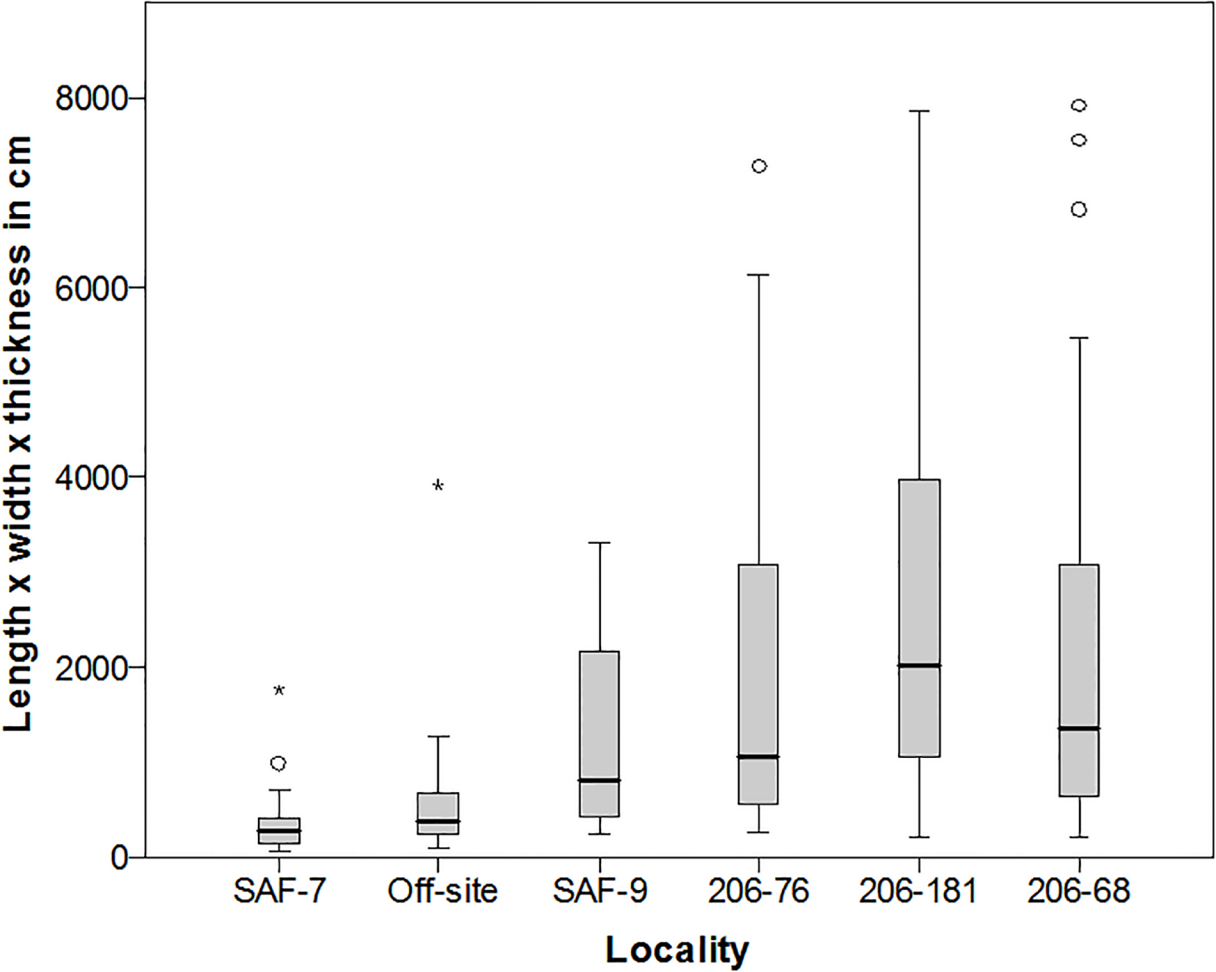
Boxplot of estimated core volume for selected Saffaqah localities, showing that localities associated with the dyke have the largest cores. Note that the very largest cores from 206–76 are too large to show at this scale.

**Fig 8:**
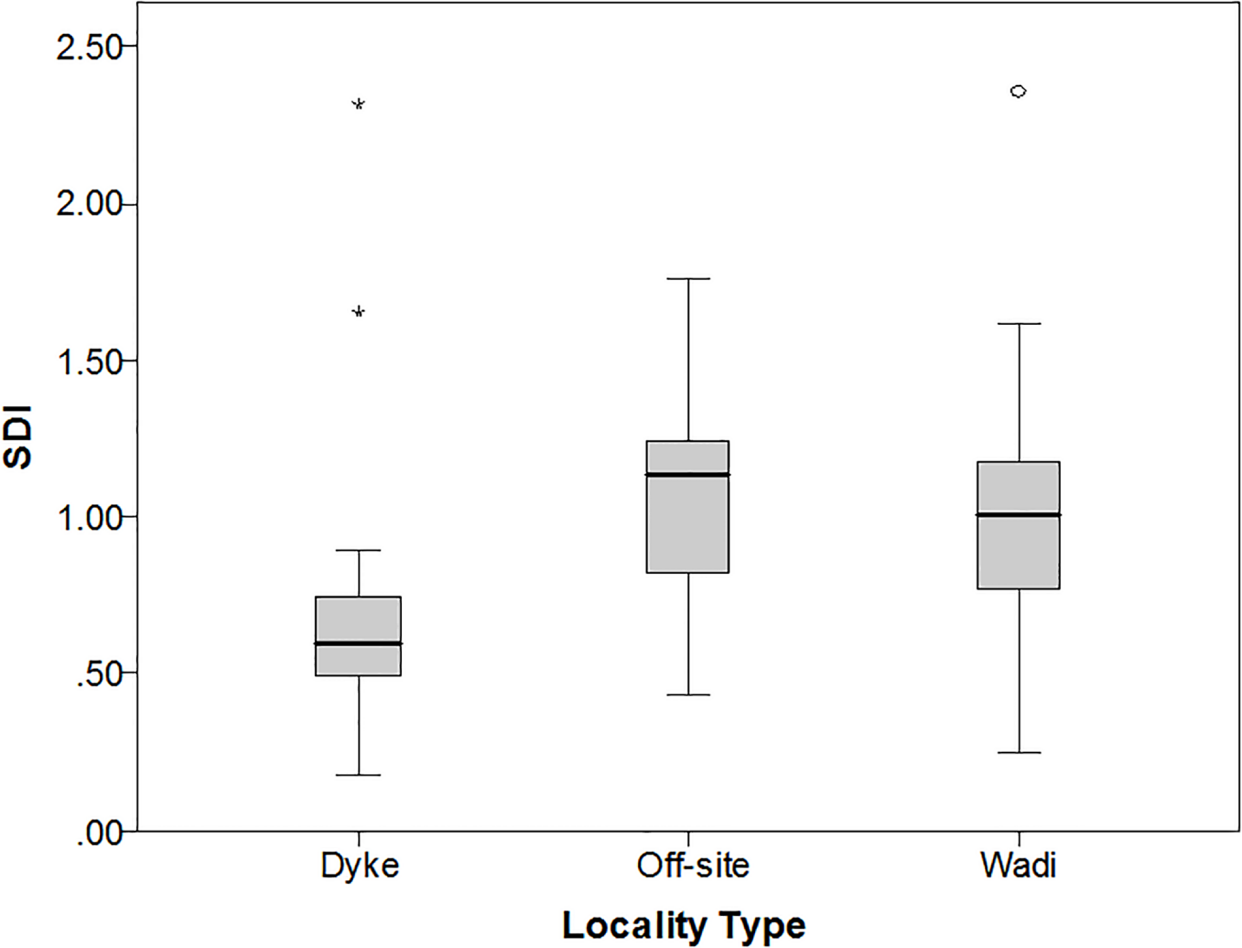
Boxplot of the scar density index (scars per square inch) for a sample of 84 Saffaqah bifaces (including early stage pieces), showing that bifaces away from the dyke are more reduced than those near the dykes.

**Fig 9:**
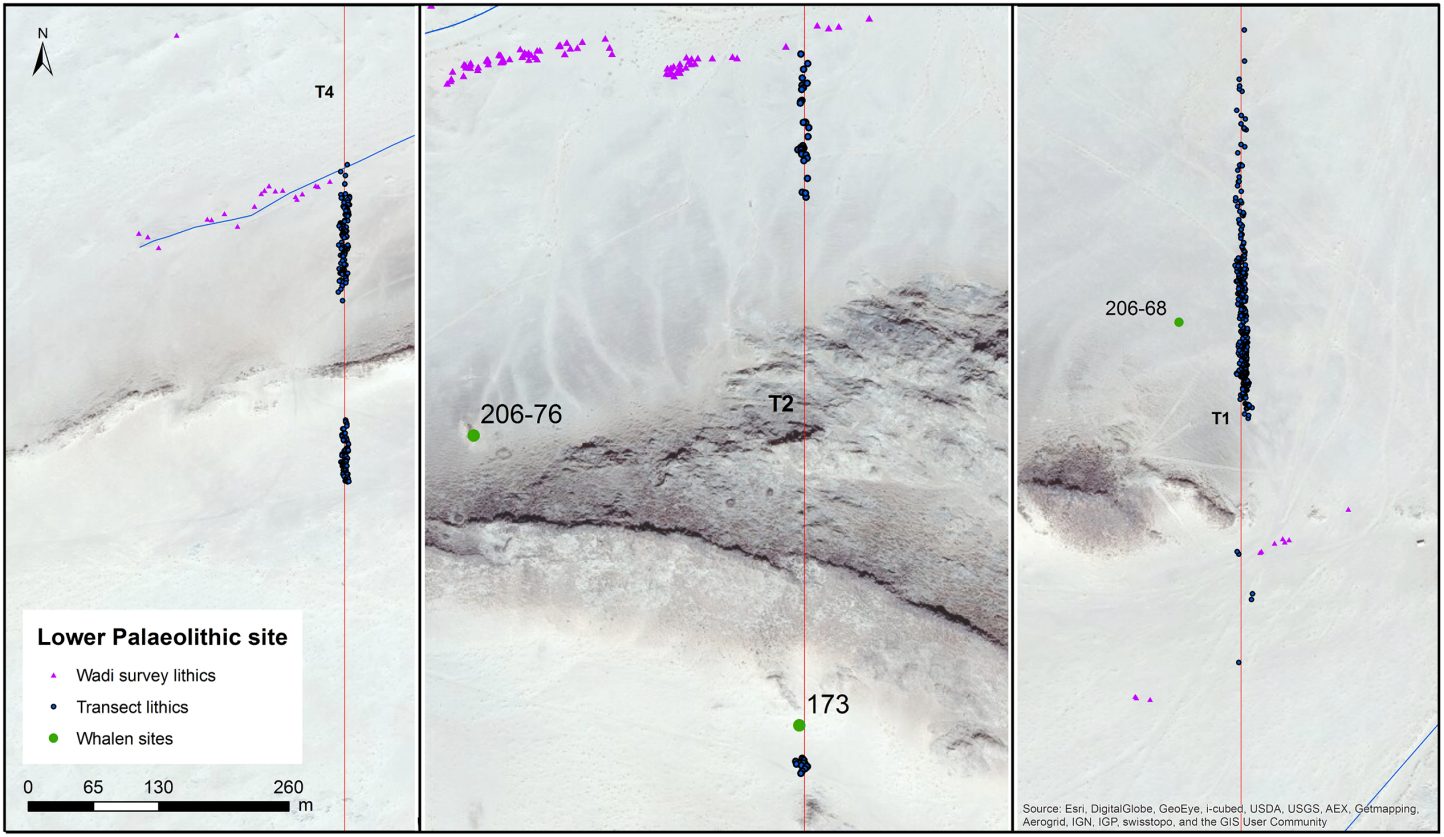
The transects crossing the Saffaqah dyke at, from left to right, the western, central, and eastern portions of the dyke. Note that artefacts stop abruptly on the approach to the dyke. Sites where a wadi flowed past a dyke were classed as dyke sites. Base layers reprinted with permission from Esri, ArcGIS, DigitalGlobe, GeoEye, i-cubed, USDA, USGS, Aex, Getmapping, Aerogrid, IGN, IGP swisstopo, and the GIS User Community under a CC-BY license, original copyright 2018.

**Fig 10:**
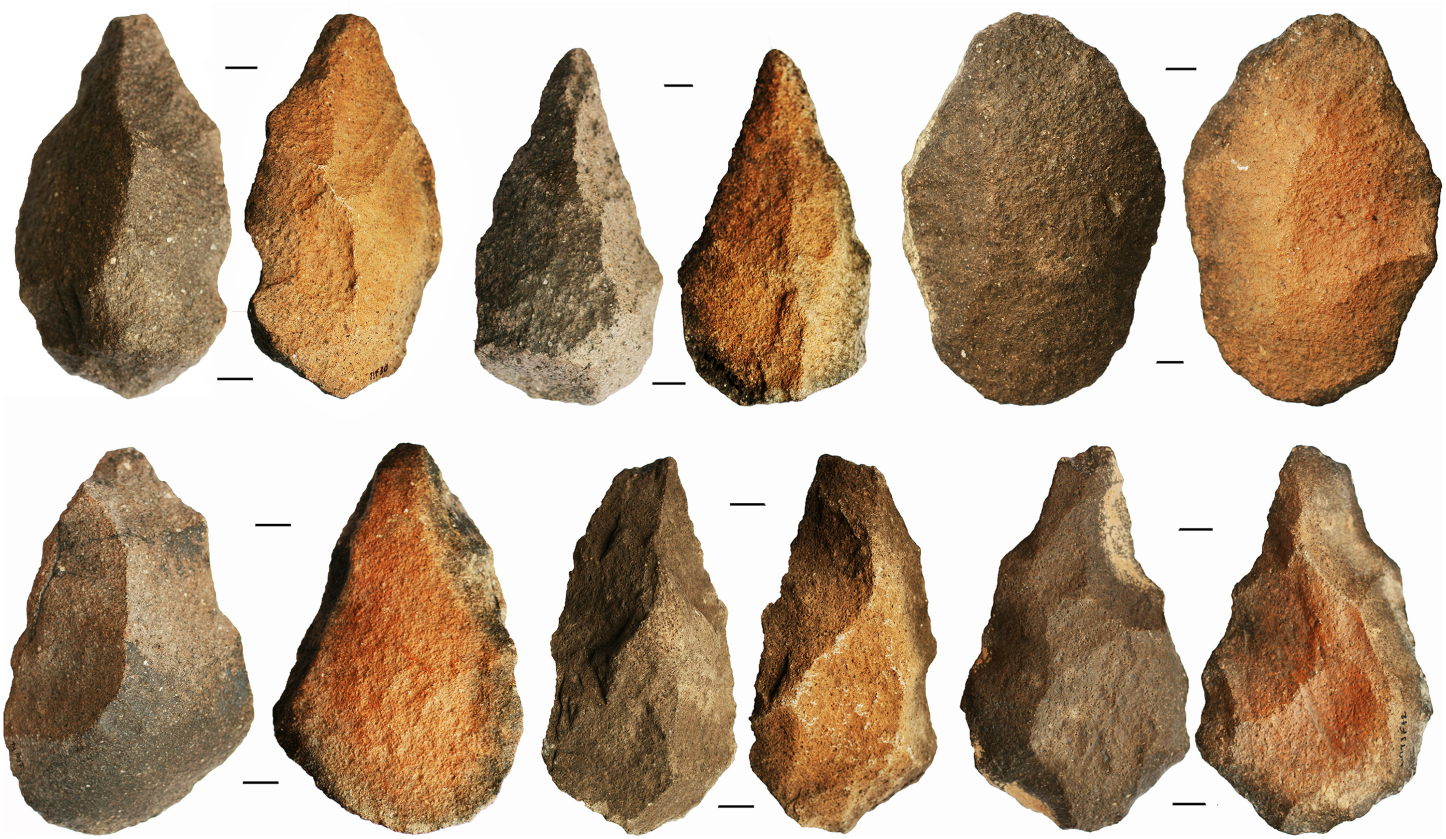
A selection of bifaces from Saffaqah showing pale purple to dark brownish grey patination on the upper exposed surface, and orange patination on the lower protected surface.

**Fig 11:**
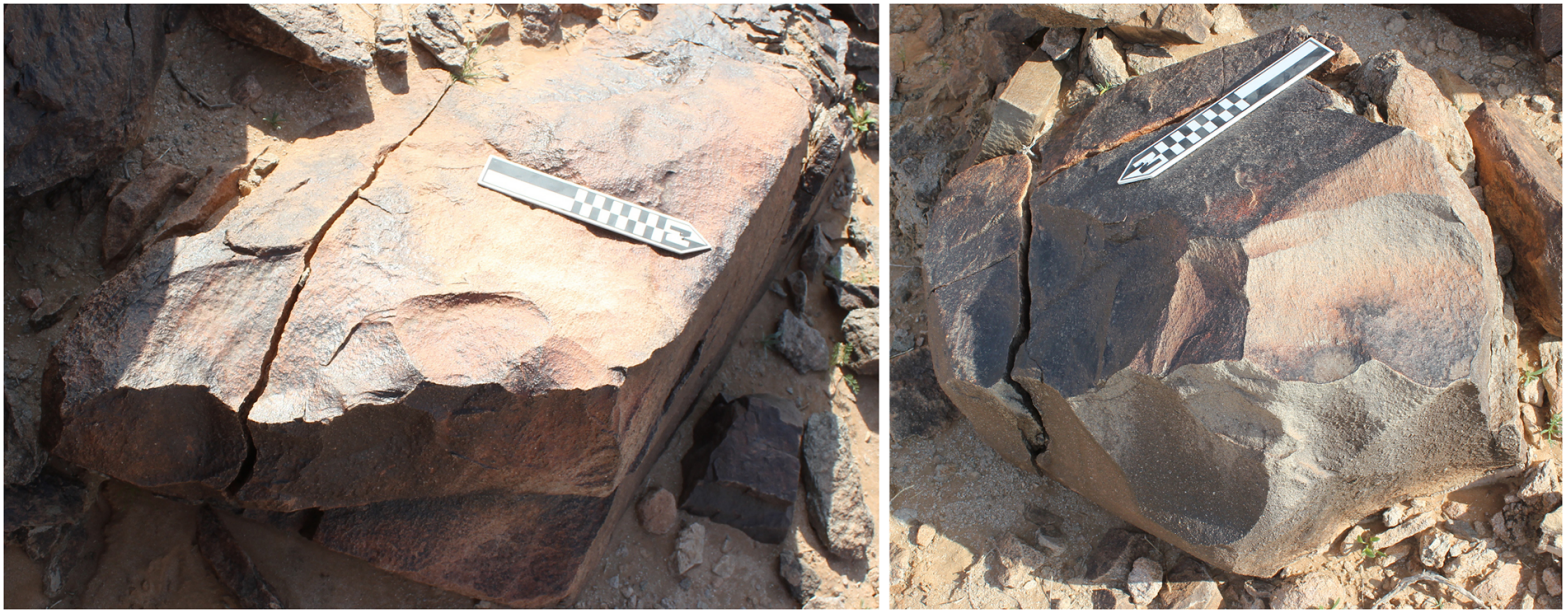
Two large blocks of andesite at the base of the dyke slope used as giant bifacial cores at Saffaqah 206–76.
